# The Multidrug Resistance 1 Gene *Abcb1* in Brain and Placenta: Comparative Analysis in Human and Guinea Pig

**DOI:** 10.1371/journal.pone.0111135

**Published:** 2014-10-29

**Authors:** Jane J. Pappas, Sophie Petropoulos, Matthew Suderman, Majid Iqbal, Vasilis Moisiadis, Gustavo Turecki, Stephen G. Matthews, Moshe Szyf

**Affiliations:** 1 Department of Pharmacology and Therapeutics, McGill University, Montreal, Canada; 2 Department of Physiology, University of Toronto, Toronto, Canada; 3 Sackler Program for Epigenetics and Psychobiology and McGill Centre for Bioinformatics, McGill University, Montreal, Quebec, Canada; 4 Departments of Psychiatry, Human Genetics and Neurology & Neurosurgery, McGill University, Montreal, Canada; 5 Departments of Obstetrics and Gynecology and Medicine, University of Toronto, Toronto, Canada; National Center for Toxicological Research, US Food and Drug Administration , United States of America

## Abstract

The Multidrug Resistance 1 (*MDR1;* alternatively *ABCB1*) gene product P-glycoprotein (P-gp), an ATP binding cassette transporter, extrudes multiple endogenous and exogenous substrates from the cell, playing an important role in normal physiology and xenobiotic distribution and bioavailability. To date, the predominant animal models used to investigate the role of P-gp have been the mouse and rat, which have two distinct genes, *Abcb1a* and *Abcb1b.* In contrast, the human has a single gene, *ABCB1,* for which only a single isoform has been validated. We and others have previously shown important differences between Abcb1a and Abcb1b, limiting the extrapolation from rodent findings to the human. Since the guinea pig has a relatively long gestation, hemomonochorial placentation and neuroanatomically mature offspring, it is more similar to the human, and may provide a more comparable model for investigating the regulation of P-gp in the brain and placenta, however, to date, the *Abcb1* gene in the guinea pig remains to be characterized. The placenta and fetal brain are barrier sites that express P-gp and that play a critical role of protection of the fetus and the fetal brain from maternally administered drugs and other xenobiotics. Using RNA sequencing (RNA-seq), reverse transcription-polymerase chain reaction (RT-PCR) and quantitative PCR (QPCR) to sequence the expressed isoforms of guinea pig *Abcb1*, we demonstrate that like the human, the guinea pig genome contains one gene for *Abcb1* but that it is expressed as at least three different isoforms via alternative splicing and alternate exon usage. Further, we demonstrate that these isoforms are more closely related to human than to rat or mouse isoforms. This striking, overall similarity and evolutionary relatedness between guinea pig *Abcb1* and human *ABCB1* indicate that the guinea pig represents a relevant animal model for investigating the function and regulation of P-gp in the placenta and brain.

## Introduction

Up to 96% of pregnant women are administered at least one prescription drug during gestation. Where the fetus is not the intended target of treatment, there is significant risk of drug exposure in the developing fetus [1], [2]. Blood-barrier sites such as the placenta and the fetal blood brain barrier (BBB) play a key role in fetal protection. The placenta is the primary barrier between maternal and fetal circulations. Multidrug Resistance 1 (MDR1; alternatively ABCB1 or P-glycoprotein (P-gp)) has been shown to limit the transplacental passage of substrates (endogenous and exogenous) into the fetal compartment and as such protects the fetus from potentially harmful compounds present in the maternal circulation [3]–[5]. Another level of fetal protection is found at the BBB. Similar to the placenta, Abcb1 in endothelial cells of the brain microvasculature limits the passage of substrates across the BBB and therefore minimizes the entry of substrates into the fetal brain [6], [7].

In most mammalian species including human, P-gp is generated from a single gene, whereas in many rodent species including the hamster, rat and mouse, two genes, *Abcb1a* and *Abcb1b*, encode distinct P-gp proteins. These exhibit overlapping and distinct substrate specificity and organ distribution [8], resulting in tissue-specific functions that bear similarity to those of human ABCB1. Nevertheless, many differences regarding expression and function of Abcb1a and Abcb1b are known. For example, Abcb1a is primarily expressed in the brain, whereas Abcb1b is primarily expressed in the placenta [9]. Moreover, mouse *Abcb1a* and *Abcb1b* promoters share minimal homology with that of the human *ABCB1* promoter, suggesting alternative transcriptional regulation [10], [11].

Studies examining the function of P-gp in the human are limited due to low availability of normal tissues for functional studies. Rodents (rats and mice) are currently widely used to examine the expression and function of P-gp *in vivo*. However, given the reported differences between the rodent and human with regard to ABCB1, these *in vivo* models are not optimal. The guinea pig represents an excellent model in which to examine the role and regulation of Abcb1 in placenta and developing BBB, *in vivo* and *in vitro.* The guinea pig has a relatively long gestation (approximately 70 days), it has hemomonochorial placentation and it gives birth to neuroanatomically mature offspring. However, the guinea pig *Abcb1* gene has not been previously characterized in detail. In the present study, we aimed to identify the guinea pig *Abcb1* gene(s). The identification of a single *Abcb1* gene in the guinea pig, as in the human, indicates that this species may provide a powerful model to screen xenobiotics and gain insight into the regulation of Abcb1, particularly in the context of development.

## Materials and Methods

### Animals, tissue collections and ethics statements

Female guinea pigs (Hartley strain, Charles River Canada, St-Constant, Quebec, Canada) were housed and bred in our animal facility as previously described [12], [13]. All studies were performed according to protocols approved by the Animal Care Committee of the University of Toronto in accordance with the Canadian Council for Animal Care. Pregnant guinea pigs were anesthetized using isofluorane (Baxter Corp., Mississauga, Ontario, Canada) and euthanized by decapitation on gestational day 50 (GD50; for placental collection; term ∼68 days) or allowed to deliver at term (for brain collection). Placentae from separate litters (n = 3) were collected and immediately frozen. Brains were isolated from postnatal day 14 (PND14) animals; euthanasia by decapitation following isofluorane inhalation followed. Brains (n = 2) were placed on ice-cold Medium 199 (LifeTechnologies, Carlsbad, California, USA) and microvessels (MVs) extracted as we have described previously [14].

Human term placental samples (n = 4; 37–39 weeks of gestation) were collected by the Mount Sinai Hospital Research Centre for Women's and Infants' Health, RCWIH BioBank (Toronto, Ontario, Canada). Collections were performed following guidelines found in “Standard Operating Procedures in the Collection of Perinatal Specimens” (available online at http://biobank.lunenfeld.ca) and informed consent was written. Tissue samples were stored at −80°C.

All studies were performed according to protocols approved by the Mount Sinai Hospital, the University of Toronto and the McGill University Institutional Review Boards. Regarding human brain samples, in all cases, family members or next of kin of the deceased subjects provided signed consent. Ethics approval nos. are: 26573 (animals), A02-M16-12B (human placentae) and A07-M76-13A (human brain).

### RNA extraction

Total RNA was extracted from guinea pig placental and brain MV and human placental samples (25–50 mg/sample) using TRIzol (LifeTechnologies) as per the modified TRIzol protocol [15]. RNA pellets were resuspended in RNase/DNase-free water (LifeTechnologies) or in Millipore water and stored at −80°C. RNA quantity and purity was assessed using NanoDrop ND 1000 spectrophotometry and 1% gel electrophoresis. RNA samples for massively parallel RNA sequencing (RNA-seq) underwent further bioanalysis to establish satisfactory quality at the Genome Quebec and McGill University Innovation Centre (Montreal, Quebec, Canada). The minimal RNA integrity value for placental and brain MV RNA used for RNA-seq was 6.1.

Human brain RNA samples (n = 4, 1–2 µg each), isolated from Brodmann's Area-18 (BA18)-enriched occipital cell samples prepared from the occipital regions of healthy young (15–26 years of age) men having committed suicide, were kindly provided by Dr. Gustavo Turecki (Montreal Neurological Institute, McGill University, MUHC, Montreal, Canada). Brain RNA samples were stored at −80°C.

### RNA-seq

RNA-seq, selected for its high-throughput capacity and base-level resolution, was undertaken at Genome Quebec and McGill University Innovation Centre. Briefly, poly-A enrichment was performed using poly-T oligo-bound magnetic beads (Sera-Mag Magnetic Oligo(dT) Beads) and fragmented using divalent cations at 94°C. First strand complementary DNA (cDNA) was copied using random pd(N)_6_ primers and SuperScript II (LifeTechnologies). Second strand was copied using Phusion DNA polymerase I and RNaseH (Finnzymes, now ThermoScientific, Ottawa, Ontario, Canada) as per the manufacturer’s protocol. Fragments were end-repaired using Klenow fragment (3′ to 5′ exo minus), adenylated, ligated to adapters using T4 ligase, gel extracted using the QIAquick Gel Extraction Kit (QIAGEN) and amplified via PCR using Phusion DNA polymerase I. cDNA libraries were sequenced using the technologies available at the time (Illumina Next Generation Sequencing for placenta; IlluminaHiSeq 2000 for brain) and 108 bp paired-end reads. Paired-end reads gives additional sequence and positional information, thereby improving library and alignment efficiencies. It was therefore the method of choice since potential alternate splice junctions were to be tracked and since the guinea pig genome is not fully annotated.

RNA-seq reads from guinea pig placenta and brain samples were aligned to the Feb 2008 *Cavia porcellus* draft (Broad Institute cavPor3) using TopHatv1.3.1 (pmid: 19261174; [16]). The average RNA-seq read coverage depths for guinea pig placental and brain MV samples were 18x and 40x, respectively. Identification of probable transcripts was performed using Cufflinks software v1.0.3 using default settings [17].

### RT-PCR and QPCR validation experiments

RNA for subsequent RT-PCR and QPCR (following reverse transcription) validation experiments (below) was DNase I treated (1U DNase I per µg of RNA; Life Technologies, Carlsbad, CA) as per the manufacturer’s protocol. Samples were re-purified using column clean-up (RNeasy Kit, QIAGEN) and re-quantified using NanoDrop ND-1000 spectrophotometry and 1% agarose gel electrophoresis.

Re-purified RNA samples (1 µg) were reverse transcribed using random pd(N)_6_ primers and Avian Myeloblastosis Virus (AMV) Reverse Transcriptase (Roche, Mississauga, Ontario, Canada) as per the manufacturer's protocol. Syntheses began with an initial denaturation of the RNA in the presence of the random primers at 70°C for 10 min followed by a 5 min incubation on ice. Reverse transcription master mixes were added and pre-incubated at room temperature (10 min). Samples were then incubated (42°C, 1 h) prior to inactivation (95°C, 10 min). cDNA samples were stored at −20°C.

cDNA (∼20 ng) was used to amplify the reference *Gapdh* gene or the target *Abcb1* amplicons. Primers were then optimized for specificity and annealing temperature using a thermal cycler (Biometra, Goettingen, Germany) and standard PCR conditions (one cycle at 95°C for 10 min, 35 cycles of 95°C for 10 sec; 60°C for 10 sec, 72°C for 10 sec; and one cycle at 72°C for 10 min). The reaction mixture was composed of the following: 2 µl of cDNA, 0.5 µl each of 10 µM forward and 10 µM reverse primer, 0.5 µl of dNTP mix (10 mM each), 0.2 µl of Hot Star Taq Plus polymerase (5 U/µl), 2.5 µl of 10X buffer and 18.8 µl RNase/DNase free water (25 µl, total reaction volume). 10 µl of the reaction volume were loaded onto an appropriate density (2–3%) agarose gel for 45 min or 2 h, respectively, to confirm RT-PCR results. Gels were stained using an ethidium bromide solution (0.5 µg/ml) for 10 min, visualized and photographed using an AlphaImager and Nikon camera. Experiments were performed in triplicate.

**Figure 1:**
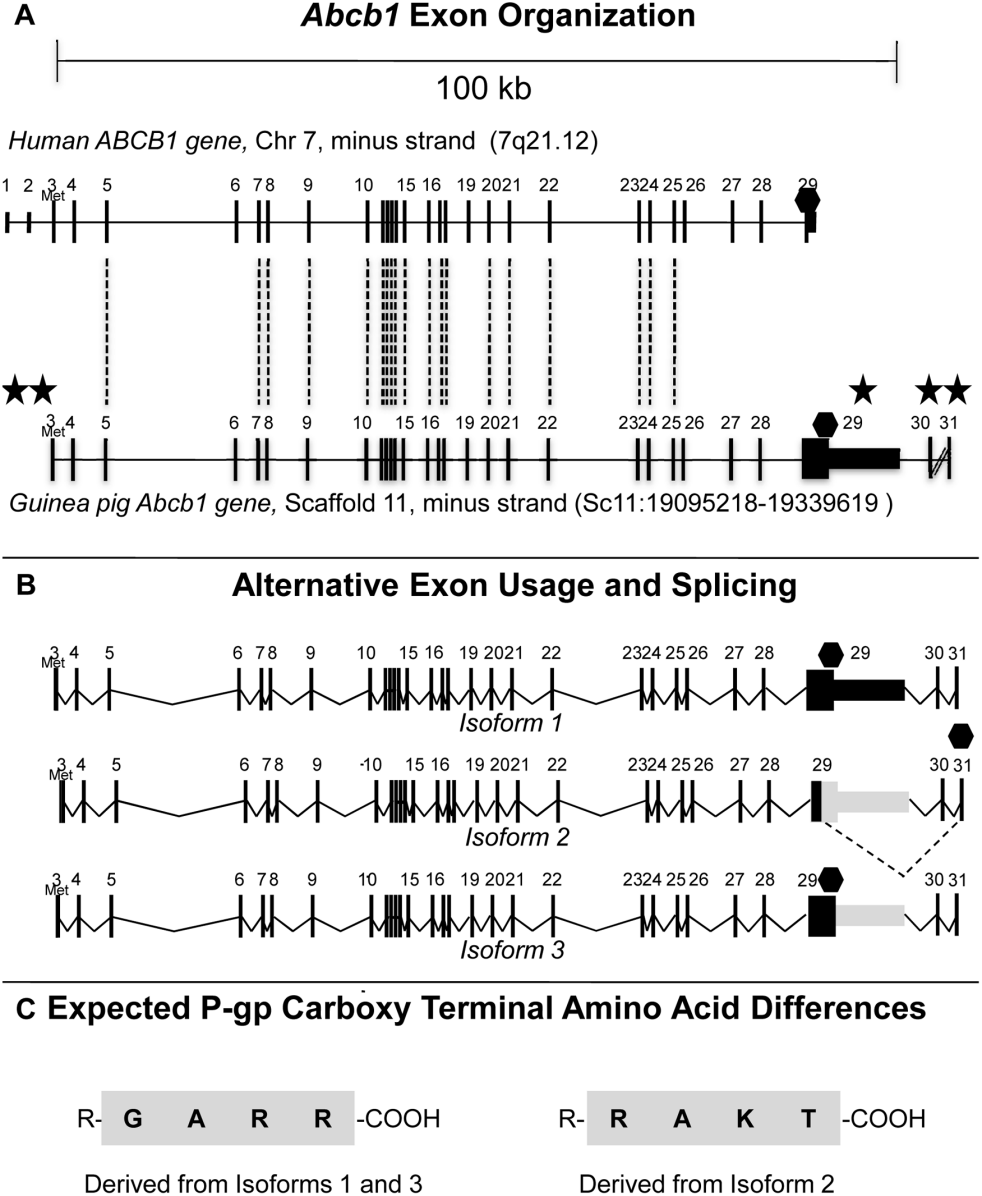
Guinea pig *Abcb1* gene, transcripts and C-terminal amino acid differences. A. Guinea pig *Abcb1* locus showing the relative localization of exons (bars) on the coding (minus) strands and in the 5′- to -3′ (left-right) orientation relative to that of the corresponding human *ABCB1* locus (above). Dashed lines join highly homologous exons. Diagram drawn to scale. Stars indicate major differences between guinea pig and human P-gp, including the absence of the first two exons, a 2.8 kb exon 29, and the presence of two additional terminal exons, exons 30 and 31. Diagonal lines between exons 30 and 31 represent a longer region than can be represented here. B. Composition of guinea pig *ABCB1* transcripts following alternate exon usage and alternative splicing. Note that exons 3 to 28 are common to all three isoforms. Dotted lines represent spliced junctions; Met represents start codon (AUG); Hexagons represent stop codons (UAA). Diagram drawn to scale. C. The 4 carboxy terminal amino acids of the guinea pig P-gp proteins according to *in silico* translation. R– = the rest of the protein; -COOH = the carboxylic acid tail. Note that diagrams based on results returned from *BLAST* or *BLAT* against the UCSC 2008 guinea pig genome assembly change periodically as the assembly evolves; hence, results regarding nucleotide sequences especially intron sizes are subject to change.

Since RNA-seq 5′ (start) reads were absent, transcripts were considered partially incomplete (Fig. S1). A diagram characterizing guinea pig *Abcb1* following corrections from validation (described below) and used to describe all following primer designs is presented in Fig. 1.

**Figure 2:**
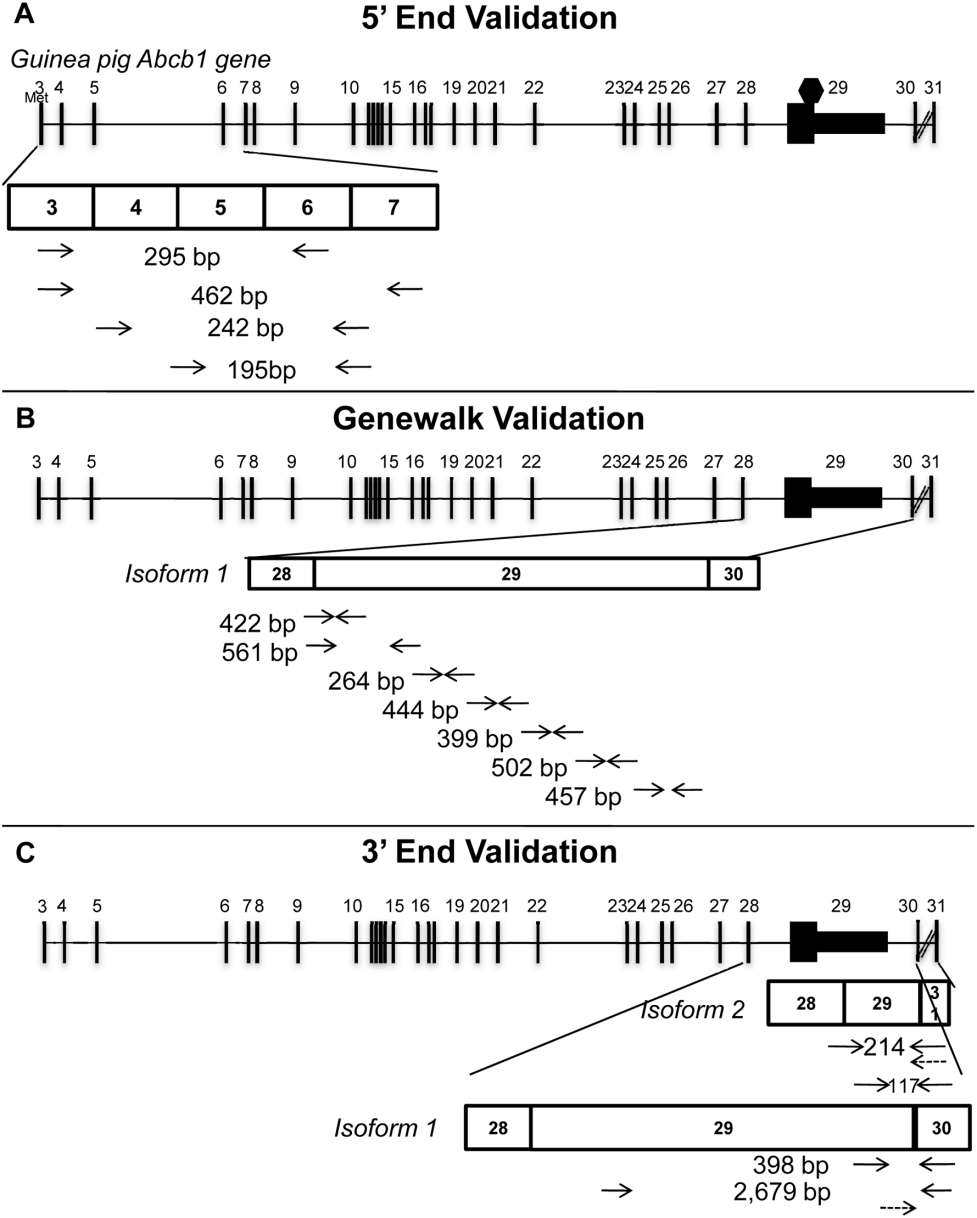
RT-PCR validation of guinea pig *Abcb1* transcripts. A. Diagram showing the relative emplacement of the primer pairs designed to identify and validate the 5′ (start) sequence via sequencing. B. Diagram showing the relative emplacement of the primer pairs designed to validate isoform 1 (genewalk). C. Diagram showing the relative emplacement of the primer pairs designed to validate the 3′ (end) sequence. Diagonal lines between exons 30 and 31 represent a longer region than can be represented here.

In order to complete the 5′ sequence of the guinea pig *Abcb1* transcripts, primers for RT-PCR were designed toward the 5′ sequence of the *Abcb1*-like mRNA complete coding DNA sequence (CDS) recently identified in guinea pig intestine (Accession no. JX312084.1), which demonstrated an additional 57 bp in the 5′ end. Primers (Fig. 2A, left and Table S1 in File S1, *5′ end validation*) were designed following basic principles (these may be found at http://www.premierbiosoft.com/tech_notes/PCR_Primer_Design.html). PCR conditions were optimized for temperature and RT-PCR products were electrophoresed on agarose gels of appropriate density, stained and photographed to confirm target specificity and lack of background noise (Fig. S3). The annealing temperature (Ta) used in all cases was 60°C.

Additional primers were designed to verify via amplification and sequencing that sequences were the same as those identified via RNA-seq: amplicons spanned exons 3–4 (containing the additional 57 bp mentioned above) or exons 4–5 (not containing the 57 bp), respectively, whereas the reverse primer was designed to span exons 6–7, common to both amplicons. (Fig. 2A, 242 and 195 bp amplicons).

RNA-seq established that exon 29 of guinea pig *Abcb1* (containing both coding sequence and the majority of the 3′-untranslated region (3′UTR)) is 2,781 bp. Given its unusually large size, an RT-PCR genewalk strategy was selected as the method of choice to validate findings. The sequences of the long terminal exon and its immediate 5′- and 3′- flanking exons were combined and used for primer design. 5′- to 3′- stepwise overlapping RT-PCR amplicons across the gene (“genewalk”) were designed (Fig. 2B) using exon junction-spanning primers when possible (e.g. 422 and 457 bp amplicons). Primer sequences and amplicon characteristics are presented in Table S1 in File S1 (*Genewalk*).

Primer design and sequences for validation of 3′ end sequences are presented in Fig. 2C and Table S1 in File S1 (*3′ end validation*). Briefly, five amplicons were designed: (1) a long 2,679 bp amplicon to validate isoform 1, where the forward is specific for the 5′ end of exon 29 and the reverse primer is specific for exon 30; (2) a shorter 398 bp amplicon to validate this same isoform but where the forward primer is specific for the 3′ end of exon 29; (3) a 214 bp amplicon to validate isoform 2 where the forward primer is specific for the 5′ end of exon 29 and the reverse primer is specific for exon 31; (4) a set of primers to confirm that no transcript results from splicing that joins a region in exon 29 present in isoform 1 to exon 31 present in isoform 2, hence this primer pair was not expected to result in a product; and (5) a 117 bp amplicon to specifically validate the splice site joining exons 29 and 31 of isoform 2. Agarose gel electrophoreses of these RT-PCR products are presented in Fig. S3.

**Figure 3:**
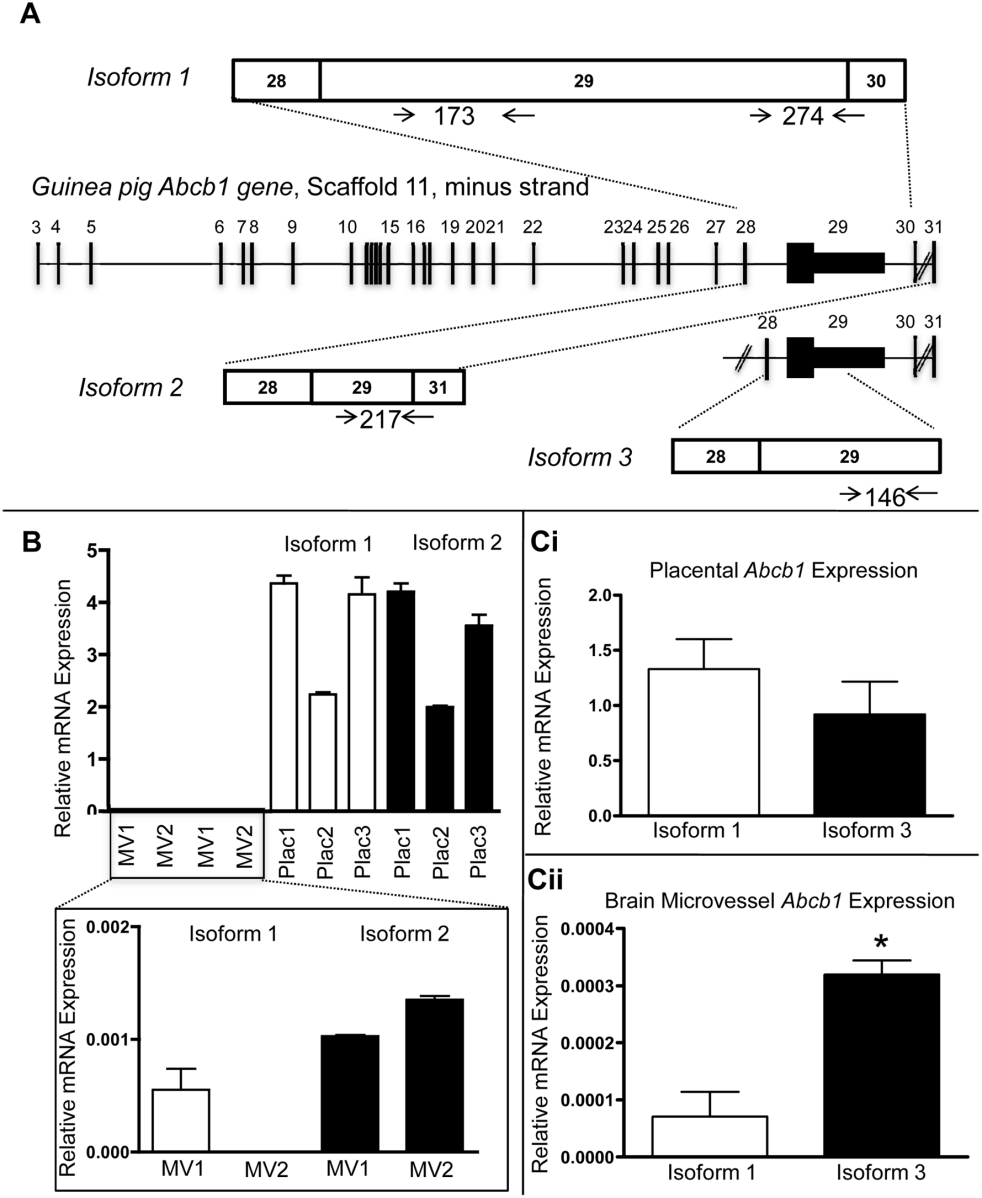
QPCR quantification of guinea pig *Abcb1* isoforms in guinea pig placenta and brain. A. Schematic diagram of QPCR primer pairs used to determine the relative abundance of *Abcb1* isoforms 1 and 2 and isoforms 1 and 3. Diagonal lines between exons 30 and 31 represent a longer region than can be represented here. B. Bar graph of relative abundance of isoforms 1 and 2 in individual placenta and brain MV samples following QPCR with primer pairs shown in A. Differences in brain magnified in inset. C. Bar graph of average relative abundance of isoforms 1 and 3 in placental samples (Ci) and brain MV samples (Cii) following QPCR with primer pairs shown in A. All bars represent mean ± SEM. *P<0.05 as determined by Student’s *t*-test.

Quantitative real-time RT-PCR (QPCR) using a LightCycler480 and accompanying software (Roche) was used to quantify isoform expression in guinea pig placental and brain MV samples. cDNA (∼20 ng) was used to amplify sequences specific to the 3′ termini of isoforms 1 or 2 using SYBR Green (Life Technologies) as per the manufacturer's protocol. In an independent experiment, isoforms 1 and 3 were quantified to determine the relative abundance of each in the placenta and brain MVs. Due to the large exon 29 in isoform 1, RT-PCR to simultaneously quantify all three isoforms was not possible. Primers (Table S1 in File S1) were designed to have the same Ta of 60°C and produce amplicons with similar amplification efficiencies (Fig. 3A). QPCR conditions were as follows: 10 min at 95°C followed by 45 cycles of 15 sec at 95°C, 30 sec at 60°C and 30 sec at 72°C. Melting curves were generated and products were electrophoresed on agarose gels and viewed using AlphaImager as described above to confirm the presence of a single band of the expected molecular weight. Statistical analyses were undertaken using Prism GraphPad and the significance was set at p<0.05.

Samples of RT-PCR products were sent to the Genome Quebec and McGill University Innovation Centre for sequencing to confirm specificity.

### Genewalk along the *ABCB1* 3′UTR in human brain and placenta

A second genewalk approach was utilized to determine whether human *ABCB1* transcripts have equally long 3′UTRs and whether they are of placental or of brain origin. The human *ABCB1* terminal exon 29 is 587 bp, of which only 204 bp of the sequence is coding, the remaining 383 bp sequence is untranslated (NM_000927.4). However, based on the information available on spliced and unspliced established sequence tags (ESTs) at the National Institutes of Health, Mammalian Gene Collection (MGC) (http://mgc.nci.nih.gov/), the exon may be well over 383 bp and approaching 1.2 kb. Primers were therefore designed to amplify stepwise overlapping sub-regions of this 1,2 kb region (Fig. S4; Table S2 in File S1).

### Protein modeling

Protein models of the amino acid sequences translated *in silico* (http://web.expasy.org/translate/) from the validated transcript sequences of guinea pig *Abcb1* isoforms as well as that of human (ABCB1) were generated using MODWEB (https://modbase.compbio.ucsf.edu/scgi/modweb.cgi; [18]) and visualized and compared following overlay using UCSF Chimera (http://www.cgl.ucsf.edu/chimera/; [19]).

### Gene synteny and phylogenetic tree construction

To compare gene organization, the following assemblies were used: Feb. 2009 human reference (GRCh37/hg19), Feb. 2008 *Cavia porcellus* draft (Broad/cavPor3), Dec. 2011 *Mus musculus* (GRCm38/mm10), Nov. 2004 *Rattus norvegicus* (Baylor 3.4/rn4) and the Chinese Hamster Genome Database (http://www.chogenome.org/).

Phylogenetic analysis, where the branch length is proportional to the number of amino acid substitutions per site, of guinea pig, mouse, rat and hamster Abcb1 proteins and human ABCB1 was performed using phylogeny.fr (http://www.phylogeny.fr/version2_cgi/simple_phylogeny.cgi; [20]).

### RT-PCR and hypothetical human *ABCB1* exons 30–32

RT-PCR was used to determine whether or not human placental or brain *ABCB1* transcripts consisting of alternatively spliced products where exon 29 is spliced to sequences representing hypothetical exons 30, 31 and 32, found using the UCSC Genome Browser (option: “Whole Cell PolyA-RNAseq Contigs Pooled from ENCODE/CSHL”), exist. RT-PCR was performed using two different anchor forward primers, one in exon 28 and another in exon 29, in combination with different reverse primers in these hypothetical exons 30, 31 and 32, on our 8 human placental and brain (Brodmann’s Area (BA18)-enriched) samples RNA samples. Primers were designed to amplify products containing one or more of these hypothetical exons (Fig. S6; Table S5 in File S1). A positive control, consisting of exon junction-spanning primers (forward primer against the junction between exons 8 and 9; reverse primer against the junction between exons 9 and 10), was used to ensure cDNA quality.

### Analysis of Human Brain *ABCB1* Transcripts from RNA-seq

RNA-seq data from 40 human brain samples derived from the hippocampus and pre-frontal cortex (BA24) from a separate study and performed according to protocols approved by the McGill University Institutional Review Board were analyzed to identify potential *ABCB1* transcripts having 3′ ends homologous to those described herein in guinea pig using Integrated Genomics Viewer 2.3 (IGV2.3).

## Results

### RNA-seq identification of one guinea pig *Abcb1* gene with three transcripts

From RNA isolated from guinea pig placental and brain microvessel (MV)-derived tissues, five transcripts were identified as potential *Abcb1* transcripts using Cufflinks software, but only three of these were found to bear significant homology to Abcb1 with multiple species including human, so only these were therefore retained for further study (Fig. S1). The two transcripts that were excluded from analysis (Fig. S2A) were built from scaffolds other than scaffold 11 and shared greater homology to other members of the ABC superfamily, more specifically *Abcb11*, *Abcb4*, and *Abcb8*. Reads for these transcripts were also on average 20-fold less abundant than other reads corresponding to *Abcb1* transcripts. One transcript (Cuff5730.1) was derived from brain MV and aligned to scaffold 3; the other transcript (Cuff10687.1) was derived from placenta and aligned to scaffold 10. Relevant sequence alignments using phylogeny.fr (http://www.phylogeny.fr/version2_cgi/simple_phylogeny.cgi; [20] are presented in Fig. S2B. The three retained transcripts (Table S3 in File S1) were all built from scaffold 11 and shared ∼87% homology with human *ABCB1* (Table 1). Of these three transcripts, two (Cuff2449.1 and Cuff2449.2) were identified from placental RNA (isoforms 1 and 2; Fig. S1) and one (Cuff10320) was identified from brain MV RNA (isoform 3; Fig. S1). Inter-transcript homology and sequence characteristics using the UCSC guinea pig 2008 genome assembly (Broad/cavPor3) established that these transcripts result from the alternative exon splicing and alternate exon usage of a single genetic locus.

**Table 1:** Percentages identity and coverage between guinea pig and human or rodent species' *Abcb1* transcripts (left) and proteins (right).

	Guinea pig *Abcb1* (transcripts)							Guinea pig P-gp (proteins)			
Transcript Accession No.	Isoform 1		Isoform 2		Isoform 3		Protein AccessionNo.	Isoform 1 & 3		Isoform 2	
	%Id	%Cov	%Id	%Cov	%Id	%Cov		%Id	%Cov	%Id	%Cov
Human *ABCB1*NM_000927.4	87	58	87	98	87	92	Human ABCB1P08183.3	86	100	86	99
Mouse *Abcb1a*NM_011076.2	84	57	84	98	84	90	Mouse Abcb1aNP_035206.2	85	97	85	97
Mouse *Abcb1b*NM_011075.2	81	57	82	98	81	90	Mouse Abcb1bNP_035205.1	79	97	79	97
Rat *Abcb1a*NM_133401.1	83	60	84	98	83	95	Rat Abcb1aNP_596892.1	85	97	85	97
Rat *Abcb1b*NM_012623.2	82	57	82	98	82	90	Rat Abcb1bNP_036755.2	80	97	80	97
Hamster *Abcb1a*NM_001243988.1	85	57	85	98	85	90	Hamster Abcb1aNP_001230917.1	85	100	85	99
Hamster *Abcb1b*NM_001243989.1	83	57	83	98	83	90	Hamster Abcb1bNP_001230918.1	79	100	79	99

%Id = Percentage Identity; %Cov = Percentage Coverage.

Absence of higher amplitude peaks at the most 5′ end and absence of a start codon (AUG) indicated that all three RNA-seq transcript sequences were missing their first exons, hereon designated exons 3 and 4 due to their high homology with human exons 3 and 4 of the placenta RNA-seq transcript or exons 3, 4 and 5 of the brain RNA-seq transcript. These 2–3 exons and start codon are known to be present in human, rodent, and nearly all other vertebrate species for which sequences have been reported. Hence, we hypothesized that our RNA-seq-derived guinea pig transcripts were incomplete at their 5′ ends (Fig. S1). A comprehensive diagram is provided in Fig. 1. This diagram was used to guide RT-PCR and QPCR primer designs toward the validation of RNA-seq-derived sequences and to identify short missing sequences.

### RT-PCR validation of guinea pig *Abcb1* sequences

Experiments in which the forward primer was designed to hybridize to the 5′ region and start codon of a guinea pig *Abcb1* transcript recently identified from guinea pig intestinal tissue (accession no. JX312084.1) succeeded in identifying the AUG codon. Two different anchoring reverse primers were used such that two amplicons of different molecular weights (295 and 462 bp amplicons, Fig. 2A, Table S1 in File S1, *5′ end validation*) were amplified (Fig. 2A). Sequencing showed that products were identical to RNA-seq and to the guinea pig genome assembly sequences except for nucleotides +1 to +57, which did not align to any reported guinea pig sequence. Sequences upstream to the AUG could not be determined as the guinea pig genome is currently incomplete in this region.

Given that a large *Abcb1* exon 29 (2,781 nt; Fig. 1A) was identified by RNA-seq but had not previously been described in the guinea pig or other species, validation was necessary. Seven sets of primers were designed to amplify stepwise overlapping regions (Fig. 2B; Table S1 in File S1, *Genewalk*). Amplicons 1–4 were specific to both placenta- and brain-derived RNA-seq sequences for exon 29, since the transcript identified in the brain (isoform 3) contains an exon sharing the first 553 of the 2,781 bp long exon contained in isoform 1. The remaining amplicons 5–7 were expected to be amplified only from placenta-derived cDNA samples. All primer sets were found to amplify the target molecular weight product in all three placental samples (example shown in Fig. S3, B). Both brain MV samples consistently amplified amplicons 1–4 (data not shown). However, one brain MV sample also amplified the remaining amplicons, consistent with the molecular weight and sequence of isoform 3, albeit at a lower intensity than that found in placenta. None of the no-reverse-transcriptase-controls were found to be positive, supporting mRNA-derived signals, not DNA-contaminants. Further RT-PCR experiments using primers flanking the border of isoform 3′s sequence confirmed that the last base of isoform 3 is nucleotide 553 of exon 29 (Fig. S3C, right).

All three *Abcb1* isoforms are comprised of exons 3 through 28 but differ at the 3′ end (exons 29, 30 and 31) due to alternate exon usage and alternative splicing with a donor splice junction in exon 29 (Fig. 1B). These differences are the basis for three differently sized mRNAs (6,527, 3,819 and 4,168 nt, for isoforms 1, 2 and 3, respectively). All isoforms have a stop codon (UAG) at position 3,817, only isoform 1 has a long 3′UTR. We first designed primers against isoform 1 and 2 (Fig. 2C; Table S1 in File S1, *3′ end validation*). Amplification of the 398 bp amplicon was possible in all samples albeit at a much lower intensity for brain MVs. Amplification of the 2.8 kb RT-PCR product was possible in all three placental samples but only in trace amounts in one of the two brain MV samples (agarose gels, Fig. S3C), consistent with previous genewalk results (Fig. S3B, right). We then designed primers against isoform 2 such that the reverse primer hybridizes to the splice acceptor site in the intron just upstream from the 24 bp terminal exon 31 (117 bp amplicon; Fig. S3D).

The results of these experiments were integrated with the RNA-seq transcripts to complete and correct the isoform sequences and have been schematized to scale in Fig. 1.

### QPCR quantification of *Abcb1* isoforms in guinea pig placenta and brain microvessels

QPCR results using isoform-specific primers for the 3′ ends of isoforms 1 and 2 (Fig. 3A, Table S1 in File S1, *Isoform quantification*) confirmed RT-PCR observations; brain MVs demonstrated trace amounts of isoforms 1 and 2 compared to the placenta (Fig. 3B). In the placenta, no significant difference was observed in relative abundance of isoform 1 and 3 suggesting similar levels of expression of all three isoforms (Fig. 3Ci). However, in the brain MVs, where overall expression levels were over 30,000-fold lower compared to the placenta, isoform 3 was approximately 4-fold higher (P<0.05) than isoform 1 (Fig. 3Cii).

### Genewalk identification of long 3′UTR of *ABCB1* in human placenta and brain

A genewalk performed across the 3′UTR of the human *ABCB1* shows that both placenta (n = 4) and brain tissues (n = 4) express *ABCB1* containing long (approximately 1.2 kb) 3′UTRs (Table S2 in File S1; Fig. S4).

### BLAST identification of homology between guinea pig *Abcb1* and human *ABCB1*

Identified mRNA transcripts were blasted against the non-redundant mRNA database as well as individually compared to human, mouse, rat, hamster and other species' *ABCB1* mRNA sequences using *NCBI Nucleotide Blast Sequence Alignment Tool* (http://blast.ncbi.nlm.nih.gov/). Guinea pig transcripts were found to bear a 3% closer identity to human *ABCB1* (NM_000927.4) than to any other species' *Abcb1*, including mouse or rat *Abcb1a* or *Abcb1b* (up to 87% identity over 98% coverage of the isoform 2 sequence, for example; Table 1, *Transcripts*). However, the differences between guinea pig and other species *ABCB1* are small, ranging between 2 to 5%; as in the case of shared identity between guinea pig and mouse transcripts, 84% with *Abcb1a* (NM_011076.2) versus 81–82% identity with *Abcb1b* (NM_011075.2). The greatest difference between isoforms stems from the very long 3′ terminal end of isoform 1.

### Differences at the C-terminus between isoforms

All three guinea pig mRNA transcripts have distinct 3′ ends resulting from alternative exon splicing and alternate exon usage (Fig. 1B). More specifically, isoforms 1 and 2, detected primarily in placenta, differ only at their 3′ ends (exons 29–31). Isoform 1 has a particularly long exon 29 (2,781 bp) and an average sized exon 30 (118 bp), while isoform 2 has an average sized exon 29 (180 bp), no exon 30, and a small exon 31 (24 bp). Hence, these three isoforms were expected to result in two proteins, because isoforms 1 and 3 utilize the same ORF and stop codon (UAG), whereas this stop codon is spliced out of isoform 2 (i.e. isoform 2 utilizes another UAG stop codon further downstream to the one utilized by isoforms 1 and 3) and consequently, a slightly different ORF. mRNA transcripts were translated *in silico* using the *Ex-Pasy Translate Tool* (http://web.expasy.org/translate/; [21]). Translation of isoforms 1 and 3 (Table S4 in File S1) results in a single protein while that of isoform 2 results in another, having a slightly different sequence. The difference stems from the last four C-terminal amino acid residues: GARR for the protein translated from isoforms 1 and 3, RAKT for the protein translated from isoform 2 (Fig. 1C), whereas the human residues are TKRQ (Fig. S5B).

The *in silico* translated guinea pig protein sequences were compared to homologous protein sequences of human, mouse, rat and other species using the *NCBI Protein Blast Sequence Alignment Tool* (http://blast.ncbi.nlm.nih.gov/). Guinea pig Abcb1 proteins share a closer identity to human ABCB1 (86%) than to mouse, rat or hamster Abcb1a or Abcb1b, however both bear a closer identity to Abcb1a from all rodent species than to Abcb1b (Table 1, *Proteins*).

This difference, affecting the last four C-terminal amino acid residues of isoforms 1–3, is both adjacent to one of the two nucleotide binding domains (NBDs) and adjacent to cytoplasmic proteins and/or plasma membrane proteins. When analyzed further using BLASTp or the Conserved Domain Architecture Retrieval Tool (cDART; http://www.ncbi.nlm.nih.gov/Structure/cdd/cdd.shtml), the last 4 amino acids (aa) are not part of a recognized domain. Rather, they are different one from another with regard to charge and pH/isoelectric point (Fig. S5C). In particular, the predicted protein product of isoform 2 has one additional uncharged amino acid (T/threonine) compared to the predicted protein of isoform 1.

### Summary of guinea pig *Abcb1* isoform characteristics

Differences due to alternative splicing and alternate exon usage are the basis for three different mRNA isoforms in the guinea pig. A summary of these differences may be found in Table 2. While isoforms 1 and 3 are highly different one from the other at the 3′ end regarding mRNA length and sequence, their sequences generate the same protein. In contrast, the placenta expresses an additional (distinct) protein, translated from isoform 2. Overall, in the placenta, isoforms 1 and 2 are predominantly expressed; in the brain, isoform 3 is predominantly expressed, although the relative MV expression level is very low.

**Table 2:** Summary of guinea pig *Abcb1* isoform characteristics. *Isoforms 1 and 3 use the same stop codon.

Isoform	Size ofmRNA (nt)	Exons 3 to 28identical	Size ofexon 29(nt)	Exon 30	Exon 31	Stopcodonposition	PolyAsequences	Predominantlyexpressed in	Protein (aa)
Isoform 1	6527	Yes	2781	Yes	No	3,817*	Yes	Placenta	1272
Isoform 2	3819	Yes	118	No	Yes	3,817	No		1272
Isoform 3	4168	Yes	553	No	No	3,817*	No	Brain	1272

### Analysis of genomic regions containing *Abcb1* genes identifies conserved synteny between human and guinea pig

**Figure 4:**
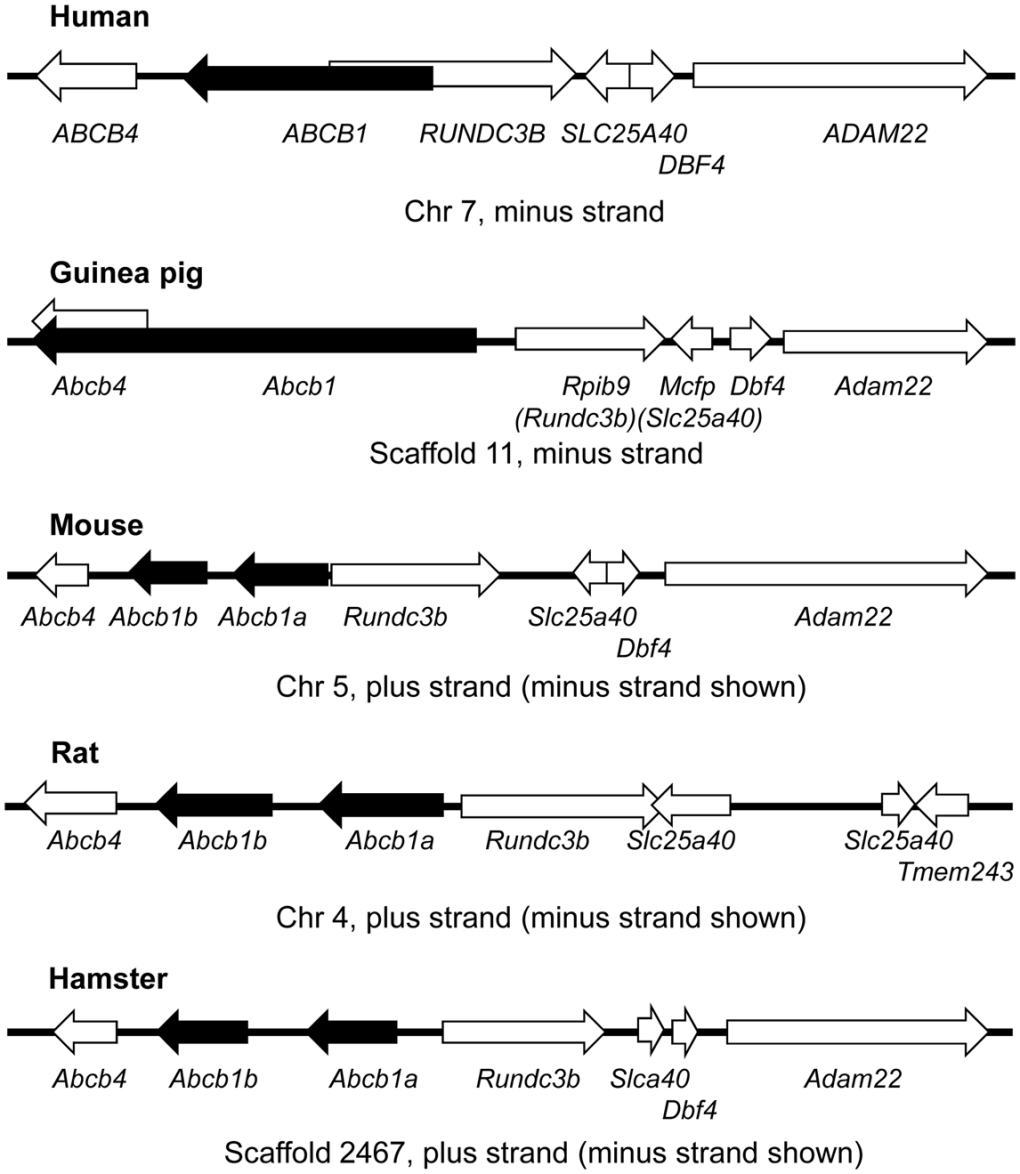
Gene synteny among guinea pig, human and rodents. Diagram of *ABCB1* and neighbouring genes in human, guinea pig, mouse, rat and hamster showing shared gene synteny, especially between guinea pig and human. The guinea pig *Abcb1* and human *ABCB1* genes are coded on the minus strand (as shown); the mouse, rat and hamster *Abcb1a* and *Abcb1b* genes are coded on the plus strand (complements shown for ease of inter-comparison). *Mcfp* and *Slc25a40* are homologs and *Rpib9* and *Rundc3b* are homologs. Figure based on the following genome assemblies: human Feb. 2009 (GRCh37/hg19), guinea pig Feb. 2008 (Broad/cavPor3), mouse Dec. 2011 (GRCm38/mm10), rat Nov. 2004 (Baylor 3.4/rn4) and Chinese Hamster Genome Database (http://www.chogenome.org/). Diagram drawn to scale.

*Abcb1* and its neighbouring loci in mouse, rat and hamster species (which are also the most frequently used for ABCB1 studies) were compared to those of the guinea pig and human. Comparisons were based on our validated guinea pig RNA-Seq data and sequence from the Feb. 2009 human (GRCh37/hg19), Dec. 2011 mouse (GRCm38/mm10), and Nov. 2004 rat (Baylor 3.4/rn4) assemblies and the Chinese Hamster Genome Database (http://www.chogenome.org/). We found differences in the number of gene loci and in their relative position to each other for rodents versus the guinea pig and the human (Fig. 4). Gene duplication has resulted in two *Abcb1* genes (*Abcb1a* and *Abcb1b*) in mouse, rat and hamster, with guinea pig, human and all other species retaining a single *Abcb1* gene.

### Phylogenetic analysis clusters guinea pig *Abcb1* and human *ABCB1* evolutions

**Figure 5:**
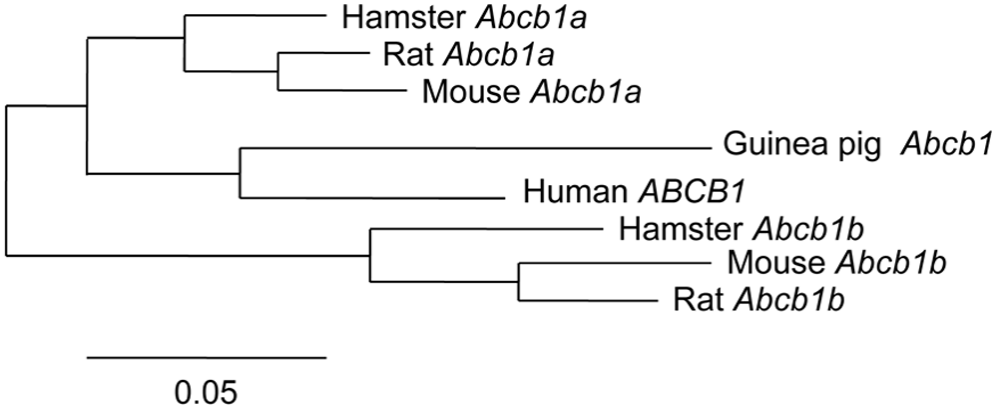
Phylogenetic tree. Phylogenetic tree of human, guinea pig, mouse, rat and hamster *ABCB1* inferring that guinea pig is closest to human and that these share a common historical evolution with *Abcb1a* of mouse, rat and hamster, whereas *Abcb1b* evolved separately. Phylogenetic tree created using http://www.phylogeny.fr/version2_cgi/simple_phylogeny.cgi and based on protein sequences. Branch length is proportional to the number of substitutions per amino acid site.

Phylogenetic analysis of Abcb1 (P-gp) proteins from guinea pig, human, mouse, rat and hamster reveals that guinea pig Abcb1 protein sequences are most closely related to human ABCB1. Further, guinea pig protein sequences cluster with Abcb1a of species known to possess two *Abcb1* genes (mouse, rat and hamster; Table 3); *Abcb1b* evolved separately (Fig. 5).

**Table 3:** Comparison of the number of *ABCB1* genes in 62 species.

ONE GENE				TWO GENES
Alpaca	Dolphin	Macaque	Shrew	**Mouse**
Anole lizard	Elephant	Marmoset	Sloth	**Rat**
Armadillo	Ferret	Medaka	Squirrel	**Hamster**
Bushbaby	Fruitfly	Megabat	Stickleback	
*C. intestinalis*	Fugu	Microbat	Tarsier	
Ciona savignyi	Gibbon	Mouse Lemur	Tasmanian devil	
C. elegans	Gorilla	Opossum	Tetraodon	
Cat	**Guinea pig**	Orangutan	Tilapia	
Chicken	Hedgehog	Panda	Tree Shrew	
Chimpanzee	Horse	Pig	Turkey	
Softshell turtle	**Human**	Pika	Wallaby	
Cod	Hyrax	Platyfish	Xenopus	
Coelacanth	Kangaroo rat	Platypus	Zebra Finch	
Cow	Lamprey	Rabbit	Zebrafish	
Dog	Hedgehog	*S. cerevisiaie*		

## Discussion

Studies described in this manuscript demonstrate that in the guinea pig, *Abcb1* exists as a single gene, akin to the human and 58 other species (among 62 species for which *Abcb1* has been annotated; Table 3). In addition, phylogenetic analysis demonstrates that guinea pig Abcb1 is more closely related to its human homolog than to its rodent homologs. These results, together with the developmental similarities between the guinea pig and the human, including long gestation, hemomonochorial placentation and neuro-anatomically mature offspring, provide strong support for the use of the guinea pig as a comparable animal model, superior to that of the rodent, toward the study of the regulation and function of ABCB1. This is important in light of the fact that rodent studies have been used extensively to extrapolate the crucial toxicological data regarding drug transfer due to the many limitations of human research.

Further, we now provide evidence that three distinct *Abcb1* mRNA isoforms are expressed in the guinea pig placenta and brain during development and that isoform expression is tissue-specific: *ABCB1* isoforms 1 and 2 are predominantly expressed in the placenta, isoform 3 is predominantly expressed in the brain. These differences may have important consequences on mRNA expression and regulation and on protein structure and predicted function.

Regarding mRNA expression and regulation, another novel finding from this study is the length (2,781 bp) of guinea pig *Abcb1* exon 29 and its correspondingly long 3′UTR, transcribed in isoform 1. Compared to isoform 2, isoform 1 requires a longer transcription time, which may result in differential temporal expression and increased cellular energy between isoforms. It may also play a role in the secondary structure and/or stability of the mRNA, potentially via polyadenylation signals or microRNA response elements (MREs) influencing post-transcriptional gene expression.

Adenylate-uridylate-rich elements (AREs) are found in the 3′UTR of many mRNAs and influence the mRNAs steady-state level by influencing degradation. The human *ABCB1* 3′UTR is AU-rich with approximately 38 AREs within a 386 bp UTR (or approximately 10%) with several of these similar to those found in c-*Myc*, c-*Fos* and *lymphokine* mRNAs, and known to cause tissue-specific RNA rapid decay [22]. Guinea pig isoform 1 (but not 2 or 3) is similar in that it also contains many AU sequences (including at least 7 AUUUA consensus sequences). While these sequences may cause the accelerated decay of isoform 1 relative to that of other isoforms, this remains to be determined. However, the human *ABCB1* 3′UTR does not behave as an active destabilizing element in HepG2 cells [22] and does not reduce the mRNA half-life in the human embryonic kidney cell line HEK293, even when the most common single nucleotide polymorphism (SNP) variants are tested *in vitro* [23], suggesting that even if these AU elements had any role in the guinea pig it is not necessarily relevant to all human tissues. It remains to be seen whether the AU elements play any regulatory role in human tissues.

MicroRNAs are known to repress expression of target genes containing microRNA response elements (MREs) within them, and MREs are most often within 3′UTRs. Very little is known regarding the conservation of MREs across species. In an attempt to discover whether the guinea pig *Abcb1* isoform 1’s 3′UTR contains MREs, the reverse complement of several human *ABCB1*-specific miRs validated to reduce *ABCB1* mRNA expression and ABCB1 activity (miR223 [24], miR508-5p [25], bta-miR145 [26], miR381 and miR-495 [27]) were searched via BLAST alignment for extrapolation to guinea pig; however, none were found. Further work is required to determine if miRs play a role in the post-transcriptional regulation of *Abcb1* in the guinea pig, with a particular emphasis on the very long 3′UTR found in this study. The human miR cluster at 14q32.31, an imprinted region, containing miR-381 and miR-495, contains over 20 miR sequences, several known to inhibit *ABCB1* in human, and there is 90% homology (BLAT) between this region and a 31 kb region in the guinea pig genome, on the plus strand of scaffold 111, therefore representing a candidate region for miR discovery.

The possibility that the long 3′UTR plays a role in the co-transcriptional translation exists, since other, smaller and silent sequence variations (SNPs translated into synonymous codons) have been shown to cause ribosomal stalling, giving nascent ABCB1 proteins greater freedom to make additional transient interactions between amino acid side chains, and resulting in the alteration of the substrate binding cavity and altered substrate specificity [28].

Regarding protein structure and function, all computational protein model predictions created using MODBASE (http://modbase.compbio.ucsf.edu/modbase-cgi/index.cgi), Swiss-Model (http://swissmodel.expasy.org/) or Phyre2 (http://www.sbg.bio.ic.ac.uk/phyre2/html/page.cgi?id=index) were partially (at least 4%) incomplete (not covering approximately 52 amino acids, minimum), and most importantly, did not cover the C-termini because X-ray crystals are not currently available for modeling the C-terminus. While resulting models, albeit incomplete (96% coverage), did show a striking similarity to known P-gp proteins (Fig. S5A), structural differences between isoforms could not be estimated. Nevertheless, given the different biochemical characteristics of the carboxy termini, it is plausible that different protein-protein interactions may be made with neighbouring proteins, either in the cytoplasm where the C-terminus bathes or within the plasma membrane. It is also possible that the RAKT sequence in Abcb1 isoform 2 is a calmodulin-dependent protein kinase II phosphorylation site, and that phosphorylation regulates isoform 2-derived protein activity, leaving the protein derived from isoform 1 unaffected [29].

There is currently limited information pertaining to the structure-function relationships regarding the C-terminus of P-gp. As such, it is difficult to speculate which P-gp characteristics (including ATP-binding, substrate specificity, rate of extrusion, coupling of substrate transport to ATP hydrolysis, signal transduction, structural flexibility, structural stability or degradation) may be affected by the differences observed in isoforms 1 and 3 compared to isoform 2.

Guinea pig *Abcb1* has two additional exons downstream to exon 29 (exons 30–31). Several lines of evidence exist pointing toward the possibility that human *ABCB1* transcripts may also be comprised of more than 29 exons. More precisely, several exons downstream to the reported 3′UTR have been observed following long RNA-seq (http://genome.ucsc.edu; Long RNA-seq track from Cold Spring Harbour Lab). In fact, human *ABCB1* reportedly has a total of 58 exons according to the European Bioinformatics Institute website (ENBL-EBI; https://www.ebi.ac.uk/). We therefore aimed to determine whether or not *ABCB1* transcripts in our 8 human placenta or brain samples contained these sequences in *ABCB1* splice products. We used RT-PCR with two different anchor forward primers, one in exon 28 and another in exon 29, in combination with reverse primers in sequences representing hypothetical exons 30, 31 and 32, found using the UCSC Genome Browser (option: “Whole Cell PolyA-RNAseq Contigs Pooled from ENCODE/CSHL”). We found no such evidence (Fig. S6, Table S5 in File S1).

No evidence of human *ABCB1* mRNA isoforms derived from brain or placenta and bearing resemblance to these novel guinea pig isoforms (i.e. variable 3′ ends due to long exon 29, alternative splicing or alternate exon usage) was found following searches conducted using multiple different databases (AceView, http://www.ncbi.nlm.nih.gov; “mRNA and EST” and “expression” tracks at UCSC genome browser Human Dec. 2013 Assembly, http://genome.ucsc.edu; Sequence Read Archive, http://www.ncbi.nlm.nih.gov/sra; Human Body Map 2.0, http://www.ensembl.info/blog/2011/05/24/human-bodymap-2-00-data-from-illumina/; DataBase of Alternative Transcripts Expression, http://bioinformatica.uniroma2.it/cgi-bin/DBATE; Alternative Splicing and Transcript Diversity database, http://www.ebi.ac.uk/astd; FastDB, http://www.fast-db.com/fastdb2/frame.html; ECgene, http://genome.ewha.ac.kr/ECgene/; Exon Intron Database, http://mcb.harvard.edu/gilbert/EID). RNA-seq data from 40 human brain samples derived from the hippocampus and pre-frontal cortex (BA24) were also visualized using Integrated Genomics Viewer 2.3 (IGV2.3), and again, no such evidence was found (data not shown). This does not necessarily negate similar functions of ABCB1 in the human and guinea pig since potential alternative transcripts in the human may be different than those found in guinea pig. Moreover, RNA-seq data from purified human brain MVs or from human placental samples enriched for the fetal component or for trophoblasts have yet to be reported.

The locus for guinea pig *Abcb4* is not currently known with certainty and may co-localize with *Abcb1* (Fig. 4). The alignment of guinea pig *Abcb* genes, a complex task due to their inherent homology, to this date remains incomplete. Currently, only one predicted (i.e. pending validation) guinea pig sequence for *Abcb4* (XM_005008654.1) has been reported on scaffold 31, not scaffold 11 where we have localized Abcb1. However, other potential loci, including the one on scaffold 11 (represented in Fig. 4) exist. It is therefore not currently possible to determine whether or not *Abcb1* and *Abcb4* co-localize at this time. Nevertheless, the last exon (exon 31) of *Abcb1* aligns to a region approximately 250 kb downstream of its 5′ end, in a region homologous to predicted *Abcb4* sequences, making the *Abcb1* locus much larger than previously thought.

In conclusion, evolution has resulted in differences between Abcb1 isoforms in the guinea pig and human. Nevertheless, overall similarity and evolutionary relatedness remain between these species. As such, the guinea pig would appear to represent a superior model for investigating Abcb1 function and regulation in comparison with other rodents. This holds in the context of human placental and brain development, and in the more general context of pharmacological, oncological, and toxicological studies. Further experimentation is required to investigate these isoforms in tissues other than the placenta and brain, and to further determine the structural and functional differences between the isoforms. Understanding regulation of Abcb1 function in the placenta and fetal blood-brain-barrier will enhance ability to protect the fetus from maternally administered therapeutics and environmental pollutants, as well as facilitate development of new drug delivery strategies for the mother and fetus.

## Supporting Information

Figure S1 **RNA-seq/Cufflinks data showing the three guinea pig** ***Abcb1*** **isoforms identified though RNA-seq.** Isoforms 1 and 2 isolated from guinea pig placenta (top) and isoform 3 isolated from guinea pig brain MVs (bottom). Stars indicate the exons missing from the initial RNA-seq data and completed following RT- and QPCR validation.
(TIF)

Figure S2 **Data showing the two guinea pig** ***Abcb1*** **candidate transcripts excluded from further study.** A. Cufflinks transcripts Cuff10687.1 (placenta) and Cuff5730 (brain MV), which bear greater homology to other ABC superfamily members than to Abcb1. B. Alignment (phylogeny.fr) showing that when compared to currently available sequences for guinea pig Abcb4 (XM_005008654.1, predicted), Abcb8 (XM_003469659.2, predicted sequence) and Abcb11 (NM_001173091.1), Cuff10687.1 and Cuff5730 align with Abcb11.
(TIF)

Figure S3 **Representative examples of guinea pig** ***Abcb1*** **RT-PCR validation products following agarose gel electrophoresis.** A. 2% agarose gel of the 295 and 462 bp RT-PCR products from the 5′ end validation. 1 ug of 100 bp ladder (FroggaBio). B. Left. 2% agarose gel of the first two of the seven genewalk products (422 and 561 bp RT-PCR products). 1 ug of 100 bp ladder. Right. Example of product (568 bp amplicon) that only amplified in one of two brain samples. 1 ug of 100 bp ladder. C. Left: 1% agarose gel of the 2.7 kb RT-PCR product. 1 ug of GeneRuler 1 kb Plus DNA Ladder (ThermoScientific). Middle: 2% agarose gel of the 398 bp product of the 3′ end validation. 1 ug of 100 bp ladder. Right: 1.5% Agarose gel of barrier RT-PCR product in brain cDNA designed such that the reverse primer R1 hybridizes to the last 20 bases of Isoform 3 cDNA (154 bp amplicon). The use of R2, as expected, does not result in a product: R2 was designed such that it hybridizes to the 20 bases downstream of R1 (this primer set would result in a 174 bp product if Isoform 3 did not end at R1 and were longer). D. 2% agarose gel of 117 bp RT-PCR product from the validation of the 3′ end. 1 ug of 100 bp ladder.
(TIF)

Figure S4 **Human** ***ABCB1*** **3′UTR genewalk.** Genewalk to confirm the presence of a long 3′UTR in human *ABCB1* transcripts in human placenta and brain. A. Diagram of the 6 overlapping amplicons. B. Representative example of an agarose gel electrophoresis of the RT-PCR products amplified from one of four placental samples taken from healthy subjects. C. Example of an agarose gel electrophoresis of the RT-PCR products amplified from one of four brain samples taken from healthy subjects. Randomly selected forward and reverse primers among those used here were paired and used to amplify longer products: products of expected molecular weights were observed (data not shown). L*.Orange Ruler DNA Ladder (ThermoScientific). RT+ = reverse transcriptase-positive samples, RT– = reverse transcriptase-negative samples. Shown are the 100 and 500 bp rungs of the Orange Ruler DNA ladder.
(TIF)

Figure S5 **Predicted protein models of guinea pig Abcb1 isoforms and comparison of carboxy terminal amino acids.** A. Protein model based on the amino acid sequence from *in silico* translation of the guinea pig ABCB1 isoform 1 or 3 sequence, primarily expressed in placenta (Ai), isoform 2, primarily expressed in brain (Aii). Models generated using Phyre2 (http://www.sbg.bio.ic.ac.uk/phyre2/html/page.cgi?id=index) B. Biochemical characteristics of the last few carboxy terminal amino acid residues. A = alanine, E = glutamic acid, G = glycine, H = histidine, K = lysine, Q = glutamine, R = arginine, T = threonine.
(TIF)

Figure S6 **Search for human** ***ABCB1*** **mRNAs with alternate 3′ ends using RT-PCR.** A. Hypothetical exons 30–32 as seen in whole cell polyA-RNAseq contigs pooled from ENCODE/CSHL, obtained at UCSC genome Browser. B. Diagram showing expected RT-PCR products if alternate human *ABCB1* mRNAs containing these sequences existed in human placenta or brain RNA. The product shown at top, with forward and reverse primers against exons 29 and 30, respectively, would contain 389 bp. The product shown in the middle, with forward and reverse primers against exons 29 and 31, respectively, would contain 1,106 bp if exon 30 is included in the transcript, or 386 bp if exon 30 is not included in the transcript. The product shown at the bottom, with forward and reverse primers against exons 29 and 32, respectively, would be 1,335 bp if exons 30 and 31 were included in the transcript, or 1,103 bp if only exon 30 was included, or 615 bp if only exon 31 was included in the transcript. C. Left. Representative agarose gel of RT-PCRs positive controls (155 bp amplicon) using placenta and brain RNA to establish RNA/cDNA integrity. Samples are: L. 1 ug of 100 bp ladder; 1. Human first trimester whole placenta; 2. Human brain-1 Brodmann's Area (BA18)-enriched sample; W. Water (no-template-control). Right. Representative agarose gel of RT-PCRs performed using brain RNA, showing that the expected product, in this case 389 bp, was not observed. Human brain Brodmann's Area (BA18)-enriched samples are: 1. Human brain 1; 2. Human brain 2; 3. Human brain 3; 4. Human brain 4; + = Reverse transcriptase positive; – = Reverse transcriptase negative control. W. No template control (Water). L. 1 ug of 100 bp ladder. Similar tests with the same results were performed using human placental RNA and for all primer pairs (data not shown). The same tests using the forward anchor primer against exon 28 rather than 29 were performed on all samples and the resulting expected products (300 bp greater in all cases), were not observed (data not shown). All RT-PCR samples were migrated twice, once as shown here and another with double the electrophoresis period to increase the resolution.
(TIF)

File S1 Table S1, RT-PCR primer pairs used in guinea pig *Abcb1* studies. Table S2, RT-PCR primer pairs used for human genewalk along the *ABCB1* 3′UTR. Table S3, Guinea pig *Abcb1* transcript coding DNA sequences (CDS). Table S4, Guinea pig Abcb1 protein sequences. Table S5, RT-PCR primer pairs used for RT-PCR on hypothetical human *ABCB1* exons 30–32.
(DOCX)
