# Supplementary figures and images for: The Multidrug Resistance 1 Gene Abcb1 in Brain and Placenta: Comparative Analysis in Human and Guinea Pig
Source: PLoS One. 2014 Oct 29;9(10):e111135. doi: 10.1371/journal.pone.0111135 (PMC4213008; doi:10.1371/journal.pone.0111135)

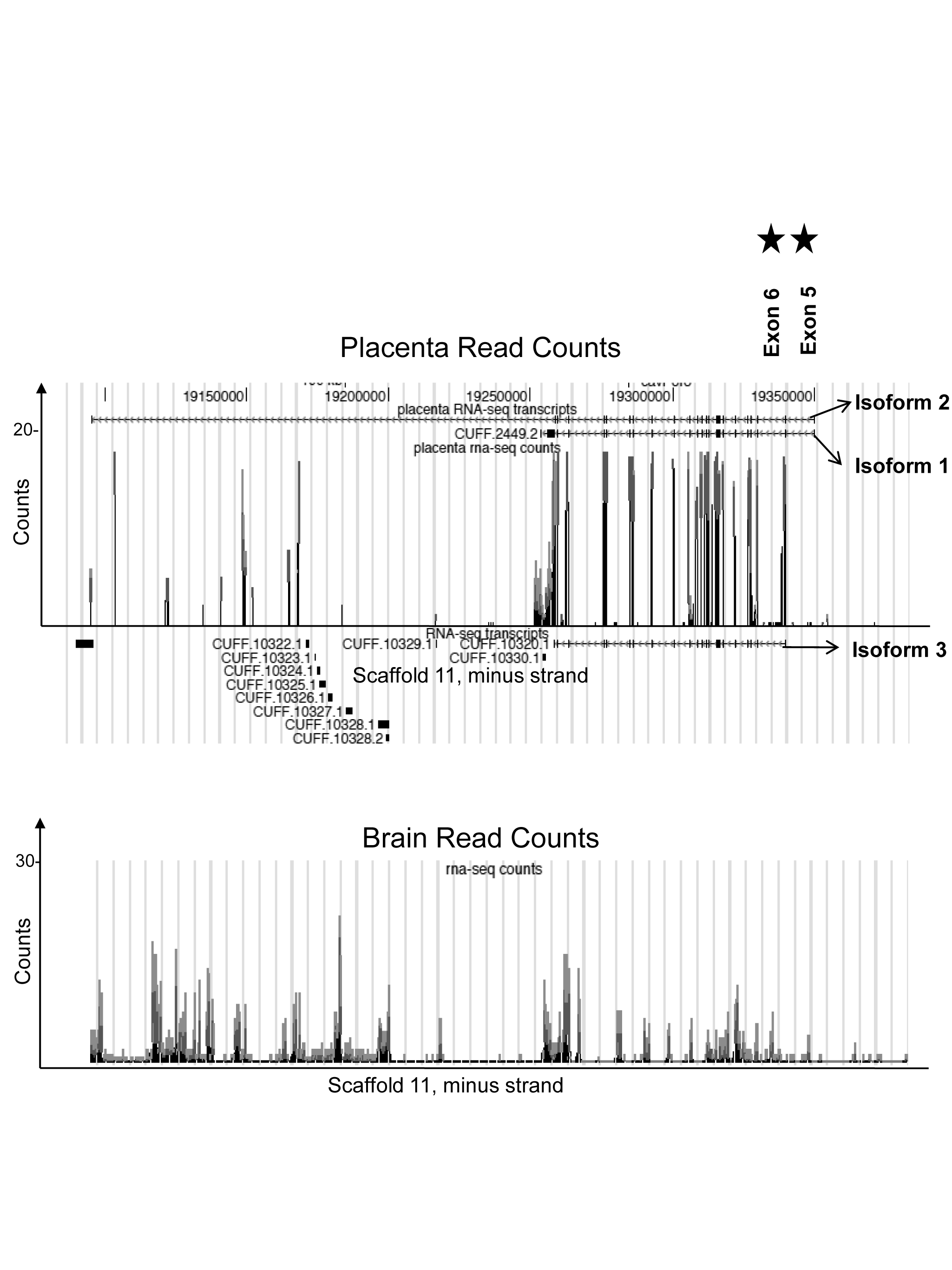

Supplement: Figure S1 — RNA-seq/Cufflinks data showing the three guinea pig Abcb1 isoforms identified though RNA-seq. Isoforms 1 and 2 isolated from guinea pig placenta (top) and isoform 3 isolated from guinea pig brain MVs (bottom). Stars indicate the exons missing from the initial RNA-seq data and completed following RT- and QPCR validation. (TIF) [file pone.0111135.s001.tif]

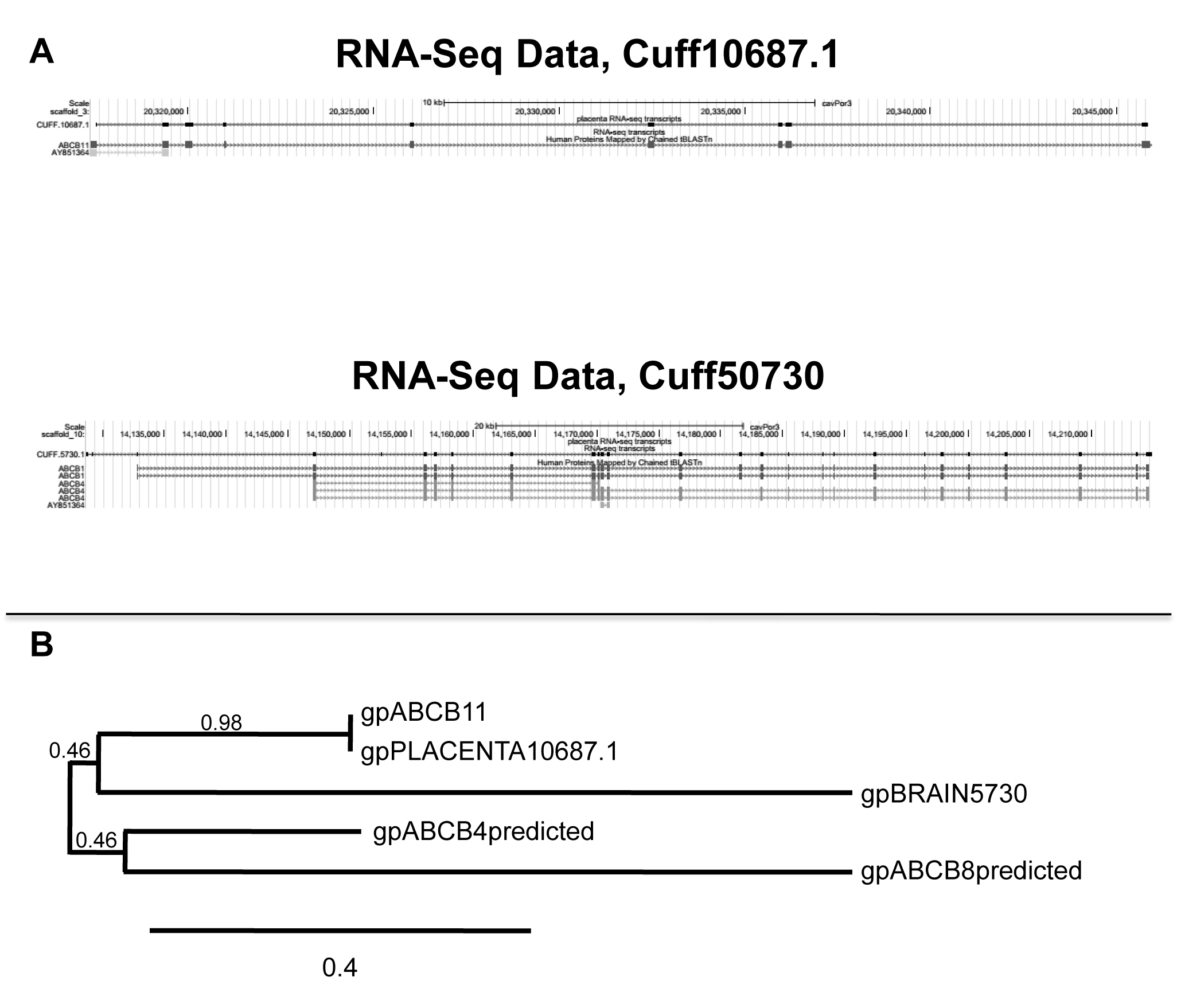

Supplement: Figure S2 — Data showing the two guinea pig Abcb1 candidate transcripts excluded from further study. A. Cufflinks transcripts Cuff10687.1 (placenta) and Cuff5730 (brain MV), which bear greater homology to other ABC superfamily members than to Abcb1. B. Alignment (phylogeny.fr) showing that when compared to currently available sequences for guinea pig Abcb4 (XM_005008654.1, predicted), Abcb8 (XM_003469659.2, predicted sequence) and Abcb11 (NM_001173091.1), Cuff10687.1 and Cuff5730 align with Abcb11. (TIF) [file pone.0111135.s002.tif]

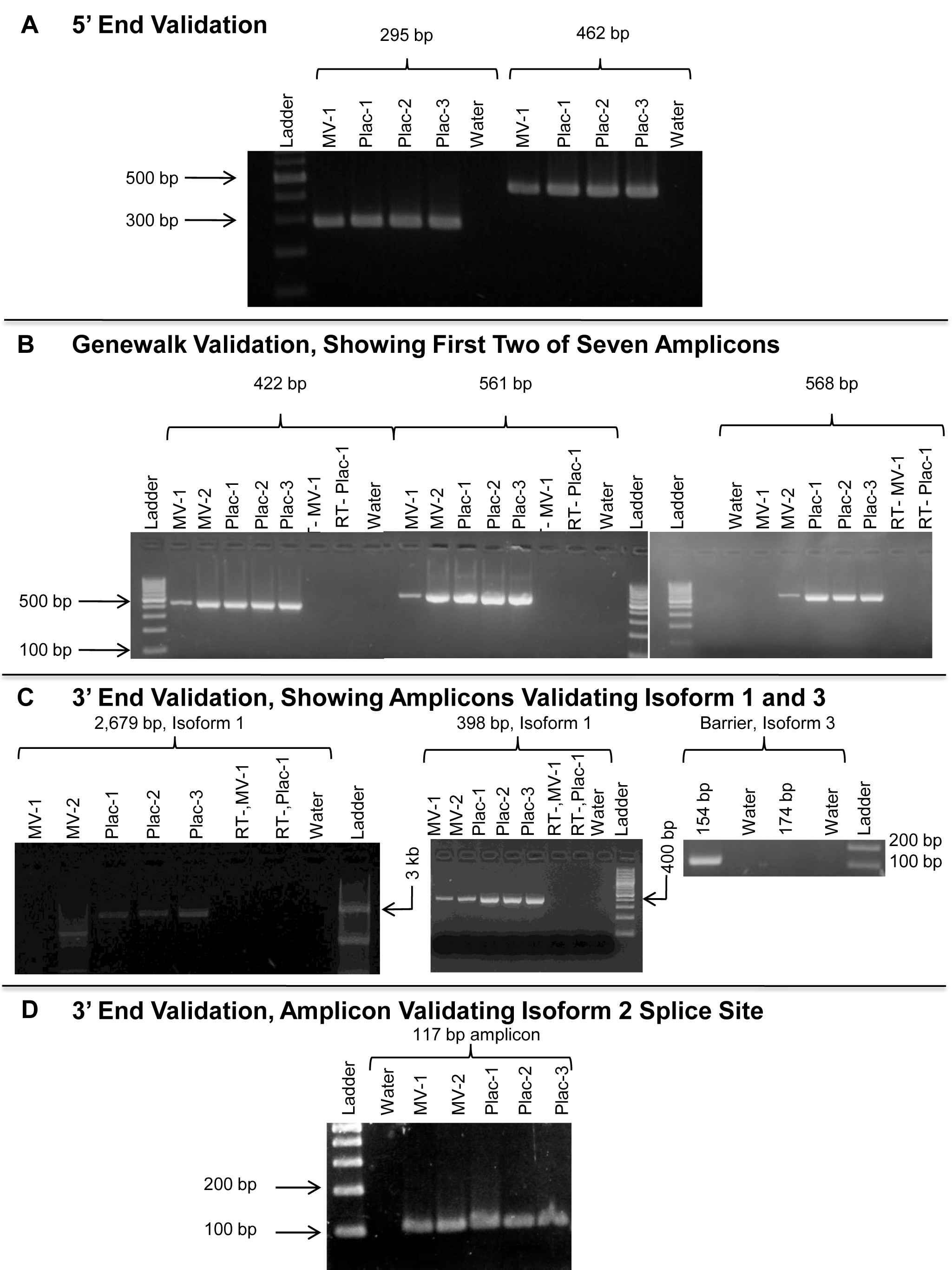

Supplement: Figure S3 — Representative examples of guinea pig Abcb1 RT-PCR validation products following agarose gel electrophoresis. A. 2% agarose gel of the 295 and 462 bp RT-PCR products from the 5′ end validation. 1 ug of 100 bp ladder (FroggaBio). B. Left. 2% agarose gel of the first two of the seven genewalk products (422 and 561 bp RT-PCR products). 1 ug of 100 bp ladder. Right. Example of product (568 bp amplicon) that only amplified in one of two brain samples. 1 ug of 100 bp ladder. C. Left: 1% agarose gel of the 2.7 kb RT-PCR product. 1 ug of GeneRuler 1 kb Plus DNA Ladder (ThermoScientific). Middle: 2% agarose gel of the 398 bp product of the 3′ end validation. 1 ug of 100 bp ladder. Right: 1.5% Agarose gel of barrier RT-PCR product in brain cDNA designed such that the reverse primer R1 hybridizes to the last 20 bases of Isoform 3 cDNA (154 bp amplicon). The use of R2, as expected, does not result in a product: R2 was designed such that it hybridizes to the 20 bases downstream of R1 (this primer set would result in a 174 bp product if Isoform 3 did not end at R1 and were longer). D. 2% agarose gel of 117 bp RT-PCR product from the validation of the 3′ end. 1 ug of 100 bp ladder. (TIF) [file pone.0111135.s003.tif]

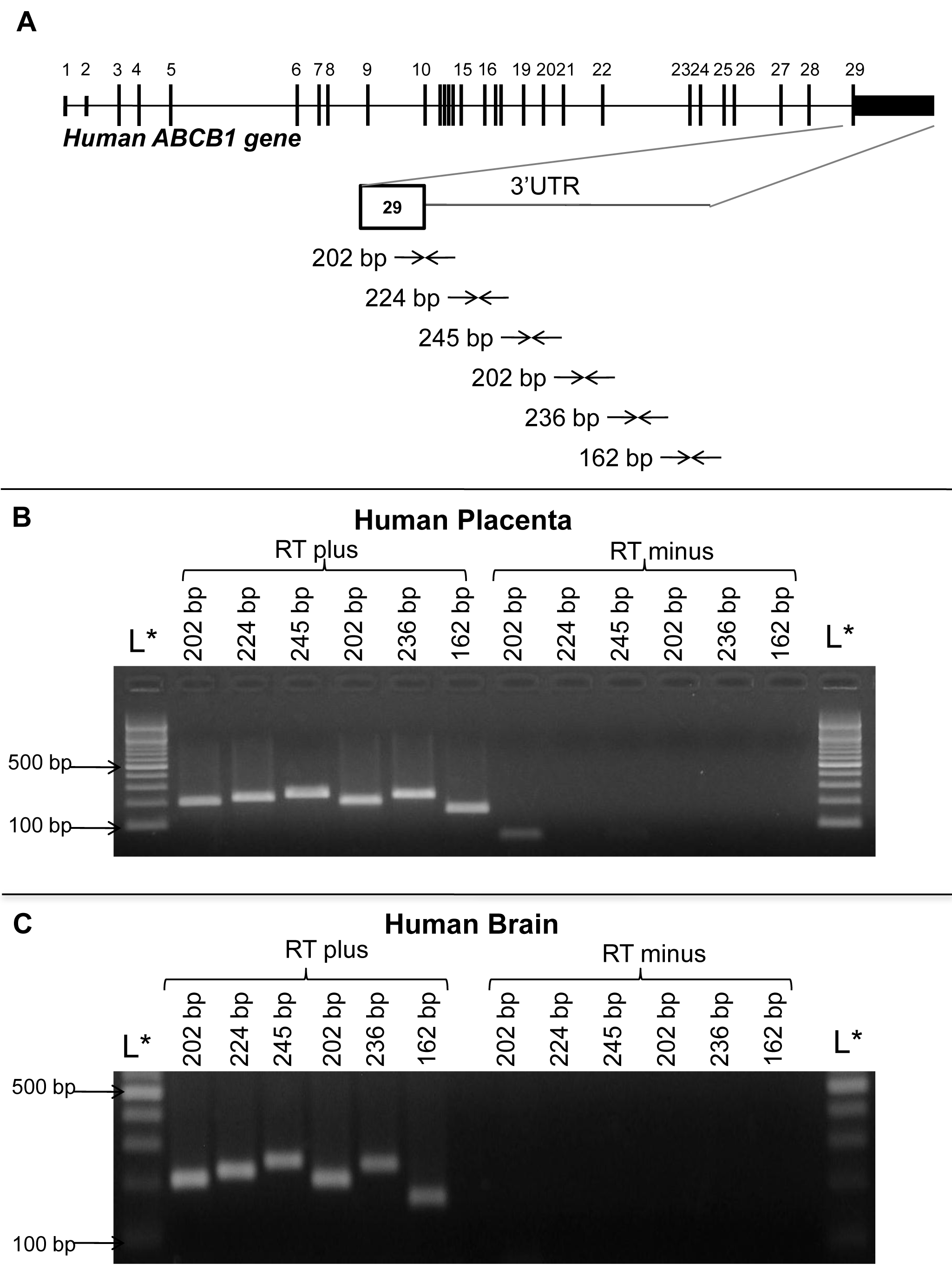

Supplement: Figure S4 — Human ABCB1 3′UTR genewalk. Genewalk to confirm the presence of a long 3′UTR in human ABCB1 transcripts in human placenta and brain. A. Diagram of the 6 overlapping amplicons. B. Representative example of an agarose gel electrophoresis of the RT-PCR products amplified from one of four placental samples taken from healthy subjects. C. Example of an agarose gel electrophoresis of the RT-PCR products amplified from one of four brain samples taken from healthy subjects. Randomly selected forward and reverse primers among those used here were paired and used to amplify longer products: products of expected molecular weights were observed (data not shown). L*.Orange Ruler DNA Ladder (ThermoScientific). RT+ = reverse transcriptase-positive samples, RT– = reverse transcriptase-negative samples. Shown are the 100 and 500 bp rungs of the Orange Ruler DNA ladder. (TIF) [file pone.0111135.s004.tif]

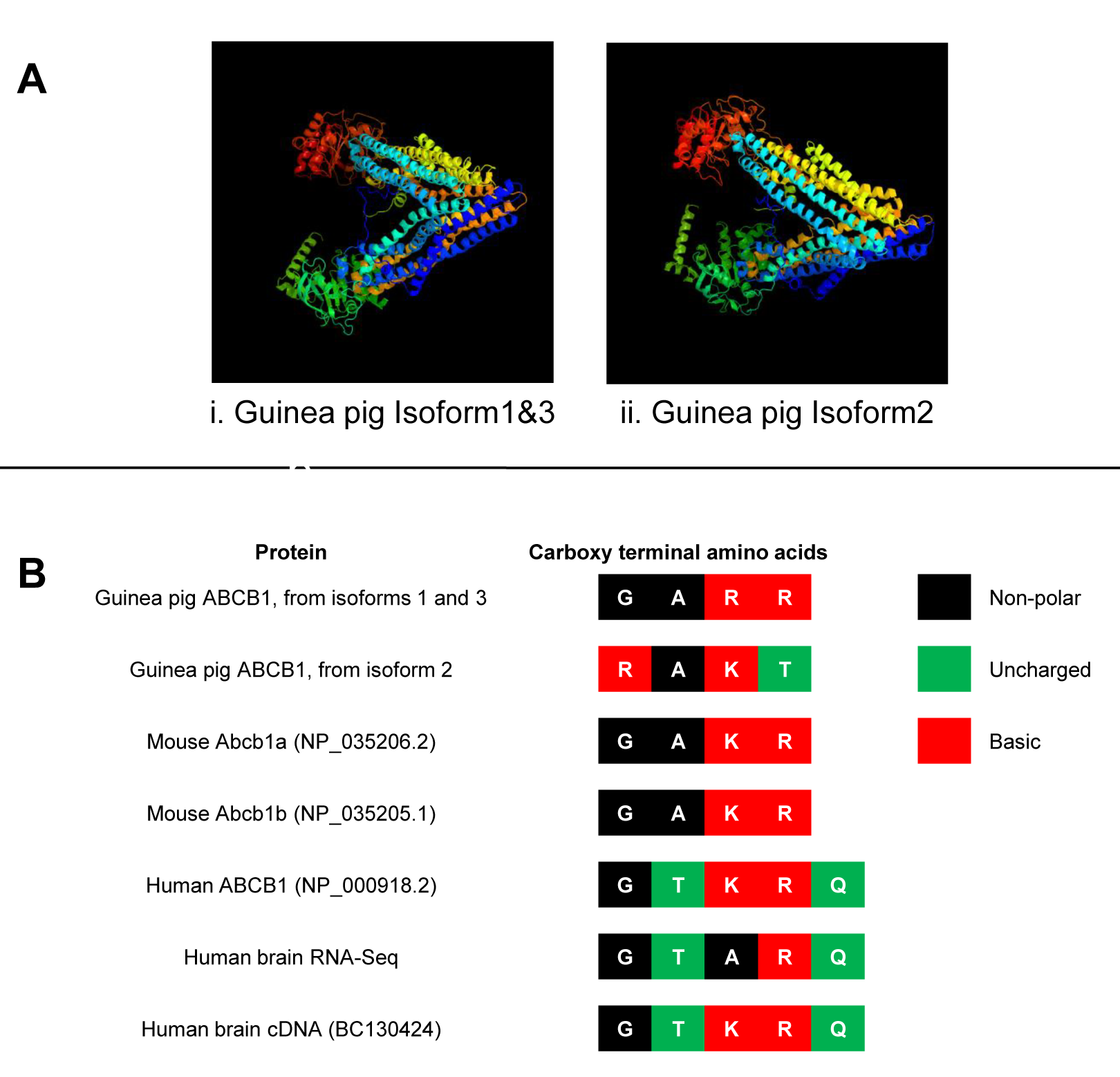

Supplement: Figure S5 — Predicted protein models of guinea pig Abcb1 isoforms and comparison of carboxy terminal amino acids. A. Protein model based on the amino acid sequence from in silico translation of the guinea pig ABCB1 isoform 1 or 3 sequence, primarily expressed in placenta (Ai), isoform 2, primarily expressed in brain (Aii). Models generated using Phyre2 (http://www.sbg.bio.ic.ac.uk/phyre2/html/page.cgi?id=index) B. Biochemical characteristics of the last few carboxy terminal amino acid residues. A = alanine, E = glutamic acid, G = glycine, H = histidine, K = lysine, Q = glutamine, R = arginine, T = threonine. (TIF) [file pone.0111135.s005.tif]

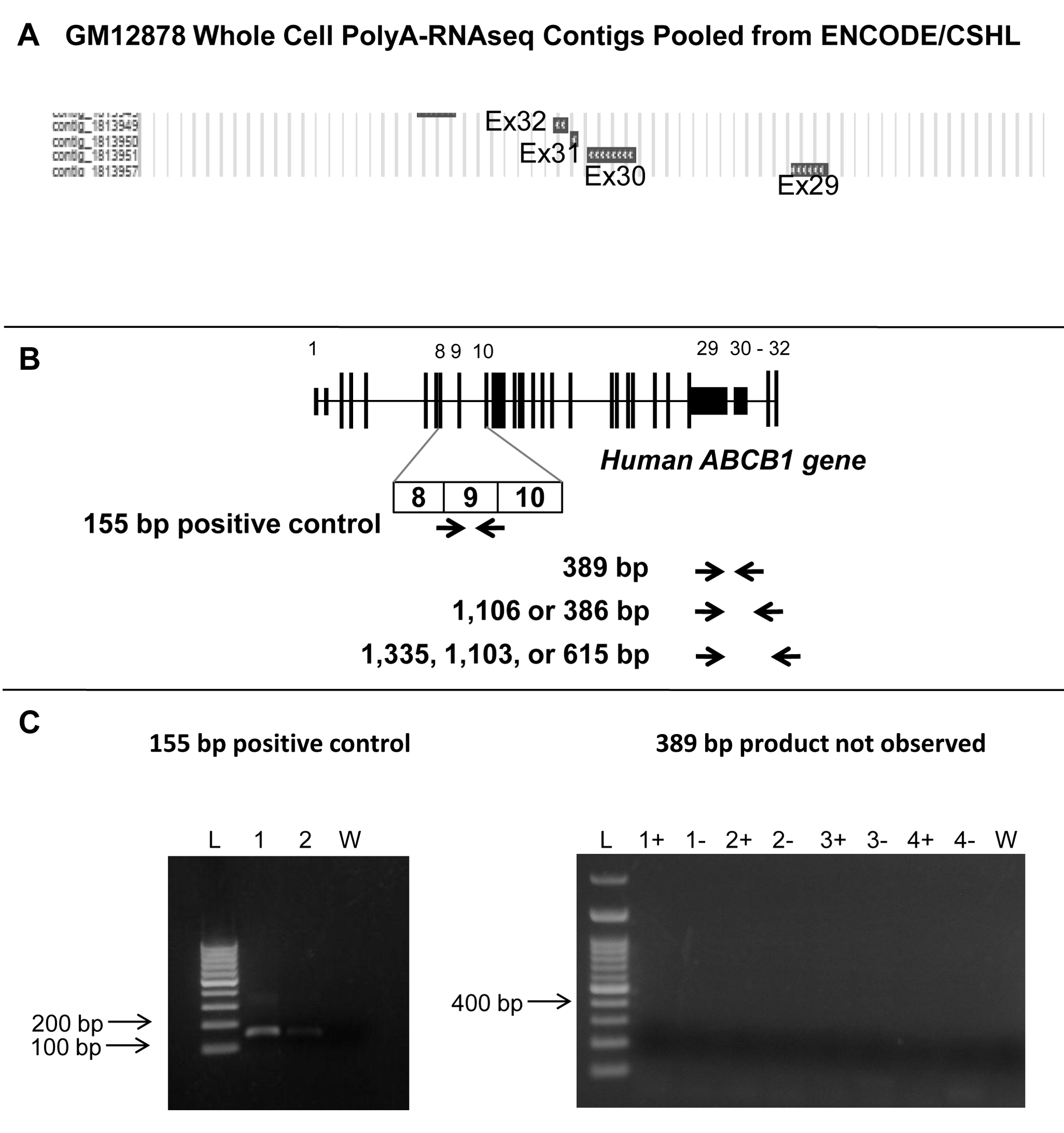

Supplement: Figure S6 — Search for human ABCB1 mRNAs with alternate 3′ ends using RT-PCR. A. Hypothetical exons 30–32 as seen in whole cell polyA-RNAseq contigs pooled from ENCODE/CSHL, obtained at UCSC genome Browser. B. Diagram showing expected RT-PCR products if alternate human ABCB1 mRNAs containing these sequences existed in human placenta or brain RNA. The product shown at top, with forward and reverse primers against exons 29 and 30, respectively, would contain 389 bp. The product shown in the middle, with forward and reverse primers against exons 29 and 31, respectively, would contain 1,106 bp if exon 30 is included in the transcript, or 386 bp if exon 30 is not included in the transcript. The product shown at the bottom, with forward and reverse primers against exons 29 and 32, respectively, would be 1,335 bp if exons 30 and 31 were included in the transcript, or 1,103 bp if only exon 30 was included, or 615 bp if only exon 31 was included in the transcript. C. Left. Representative agarose gel of RT-PCRs positive controls (155 bp amplicon) using placenta and brain RNA to establish RNA/cDNA integrity. Samples are: L. 1 ug of 100 bp ladder; 1. Human first trimester whole placenta; 2. Human brain-1 Brodmann's Area (BA18)-enriched sample; W. Water (no-template-control). Right. Representative agarose gel of RT-PCRs performed using brain RNA, showing that the expected product, in this case 389 bp, was not observed. Human brain Brodmann's Area (BA18)-enriched samples are: 1. Human brain 1; 2. Human brain 2; 3. Human brain 3; 4. Human brain 4; + = Reverse transcriptase positive; – = Reverse transcriptase negative control. W. No template control (Water). L. 1 ug of 100 bp ladder. Similar tests with the same results were performed using human placental RNA and for all primer pairs (data not shown). The same tests using the forward anchor primer against exon 28 rather than 29 were performed on all samples and the resulting expected products (300 bp greater in all cases), were not observed (d [file pone.0111135.s006.tif]
