# Supplementary material for: The Multidrug Resistance 1 Gene Abcb1 in Brain and Placenta: Comparative Analysis in Human and Guinea Pig
Source: PLoS One. 2014 Oct 29;9(10):e111135. doi: 10.1371/journal.pone.0111135 (PMC4213008; doi:10.1371/journal.pone.0111135)
Supplement: File S1 — Table S1, RT-PCR primer pairs used in guinea pig Abcb1 studies. Table S2, RT-PCR primer pairs used for human genewalk along the ABCB1 3′UTR. Table S3, Guinea pig Abcb1 transcript coding DNA sequences (CDS). Table S4, Guinea pig Abcb1 protein sequences. Table S5, RT-PCR primer pairs used for RT-PCR on hypothetical human ABCB1 exons 30–32. (DOCX) [file pone.0111135.s007.docx]

**SUPPLEMENTARY TABLES**

**Table S1.** RT-PCR primer pairs used in guinea pig *Abcb1* studies. *Primer pairs used for QPCR.

| **Primer Set** | **Primer Name** | **Primer sequence (5'-3')** | **Product MW (bp)** |
| --- | --- | --- | --- |
| **5' end validation** | | | |
| 1 | Start F1 | ATGGATCACGAAGGGGACTTC | 295 |
|  | Start R1 | CCACTGACTTCACTTTTATCTAA |  |
| 2 | Start F1 | ATGGATCACGAAGGGGACTTC | 462 |
|  | Start R2 | AGGCACCAAAATGAAACCTG |  |
| 3 | Exons3-4F | GCCATGTTTCGCTATTC | 195 |
|  | Exons4-5R | CACTGACTTCACTTTTATCTAATG |  |
| 4 | Exons3-4F | GCCATGTTTCGCTATTC | 242 |
|  | Exons5-6R | ATAATAATAGGCATACGTGGTCA |  |
| **Genewalk validation** | | | |
| 5 | GW set 1F | GTGAAAAGGTTGTCCAGGAAGCC | 422 |
|  | GW set 1R | CCATCCACTCTCCATTTGATAATGG |  |
| 6 | GW set 1F | GTGAAAAGGTTGTCCAGGAAGCC | 561 |
|  | GW set 2R | AGGCACTTTTGGCAAACATAACAGT |  |
| 7 | GW set 3F | CCATTATCAAATGGAGAGTGGATGG | 264 |
|  | GW set 3R | TCCAAGATTATACTATTCAAATTGC |  |
| 8 | GW set 4F | GCAATTTGAATAGTATAATCTTGGA | 444 |
|  | GW set 4R | GTGTTTGTCAACTCCCTCGTCG |  |
| 9 | GW set 5F | CGACGAGGGAGTTGACAAACAC | 399 |
|  | GW set 5R | ATTTGTGACTATTTACTCTTGTAATGG |  |
| 10 | GW set 6F | CCATTACAAGAGTAAATAGTCACAAAT | 502 |
|  | GW set 6R | TTTGGAAGAACATCTAGACACAAGC |  |
| 11 | GW set 7F | GCTTGTGTCTAGATGTTCTTCCAAA | 457 |
|  | GW set 7R | CATTGAAATCCTCCACTGTTACTGC |  |
| **3' end validation** | | | |
| 12 | 3' end set AF | TTTGACCACCAAGGAATAAAA | No product expected |
|  | 3' end set AR | CTGGTGTGTGCCATGTTCCT |  |
| 13 | 3' end set BF | CGCTAGTGAAGCCTGCCTAT | 2,679 |
|  | 3' end set BR | CAGCATGATACACCCACACC |  |
| 14 | 3' end set CF | CGCACATTCTTCTCATCAACA | 398 |
|  | 3' end set CR | CAGCATGATACACCCACACC |  |
| 15 | 3' end set DF | GTGAAAAGGTTGTCCAGGAAGCC | 214 |
|  | 3' end set DR | CTGGTGTGTGCCATGTTCCT |  |
| 16 | 3' end set EF | GAATGCAGACCTCATCGTGGTG | 117 |
|  | 3' end set ER | CTAGCCTGGACCCTGACCATG |  |
| **Isoform quantification** | | | |
| 17* | 1onlyF | CAGCAATCAAACAAGTATATGG | 274 |
|  | 1onlytermR | GTCCAACATAAAAATCAATC |  |
| 18* | 3' end set EF | GAATGCAGACCTCATCGTGGTG | 117 |
|  | 3' end set ER | CTAGCCTGGACCCTGACCATG |  |
| 19* | Barrier_F1 | TGGATGGTAGTTTTAATTGTATTACAG | 146 |
|  | Barrier_R1 | AGGCACTTTTGGCAAACATAACAGT |  |
| 20* | Barrier_F1 | TGGATGGTAGTTTTAATTGTATTACAG | 173 |
|  | Barrier_R2 | CAAGCATATGAAAGTGTAGTTTTATTG |  |

**Table S2.** RT-PCR primer pairs used for human genewalk along the *ABCB1* 3'UTR.

| **Set** | **Primer Name** | **Primer sequence (5'-3')** | **Product MW (bp)** |
| --- | --- | --- | --- |
| 1 | HGW_F1 | GAAGCCCTGGACAAAGCCAGAG | 202 |
|  | HGW_R1 | AGTTCACTGGCGCTTTGTTCCAG |  |
| 2 | HGW_F2 | TGGAACAAAGCGCCAGTGAACTC | 224 |
|  | HGW_R2 | CAGTTTAAACTATGATTTCTCTCCAC |  |
| 3 | HGW_F3 | GTGGAGAGAAATCATAGTTTAAACTG | 245 |
|  | HGW_R3 | TGAGAGAAGATATATTCACAGGCAG |  |
| 4 | HGW_F4 | ACTGCCTGTGAATATATCTTCTCTC | 202 |
|  | HGW_R4 | GCAAGAATCAGCAGGATCAAGTC |  |
| 5 | HGW_F5 | GACTTGATCCTGCTGATTCTTGC | 236 |
|  | HGW_R5 | TACTGGCAAAAGTGATTCATTATCAAGAC |  |
| 6 | HGW_F6 | TTGTCTTGATAATGAATCACTTTTGCCAG | 162 |
|  | HGW_R6 | GTCGTCCAGAGTCCCAACCTTTG |  |

**Table S3.** Guinea pig *Abcb1* transcript coding DNA sequences (CDS).

>Isoform 1 CDS

ATGGATCACGAAGGGGACTTCGGTGGAAAAGCAGGGGACTATAAACTGGGGAAGAAAAGT

AAAAAGGAGAAGAAGGAAAAGAAACCAACTGTTAGTACATTTGCCATGTTTCGCTATTCA

AATTGGCTGGACAGGTTGTACATGGTGCTGGGGACTCTGGCTGCTATCATCCATGGAGCT

GCGCTCCCTCTCTTGATGCTGGTGTTTGGAGACATGACAGACACCTTTGTAAATGGAAGT

GGCACAAACTCATCAAATGCATCAAGTACATTAGATAAAAGTGAAGTCAGTGGACCAGAC

AATCTGGAGGAAAAAATGACCACGTATGCCTATTATTATTCTGGAATCGGTGCTGGTGTG

CTTATTGCTGCTTACATTCAGGTTTCATTTTGGTGCCTGGCAGCTGGAAGACAGATACAC

AAAATTAGAACACAGTTTTTCCATGCAATAATGAAACAGGAGATTGGCTGGTTTGATGTG

CATGATGCTGGGGAGCTGAACACCCGCCCCACAGATGATGTCTCCAAAATTAATGAAGGA

ATTGGTGACAAAATTGGATTGTTCTTTCAATCATTGGCAACATTTTTGACTGGTTTCATA

ATAGGATTTACACGTGGTTGGAAGCTCACTCTTGTGATTTTGGCTGTCAGCCCTGTTCTT

GGACTATCAGCTGCAATCTGGGCAAAGATACTGTCTTCATTTACTGATAAAGAGCTATCA

GCATATGCAAAAGCTGGAGCCGTGGCTGAAGAAGCCTTAGCAGCCATTAGAACTGTGATT

GCATTTGGAGGACAAAGCAAAGAACTTGAAAGGTACAATAAAAATTTAGAAGAAGCTAAA

AGAATTGGAATAAAGAAAGCCATCACATCCAATATTTCTATTGGTGCTGCTTTCCTGCTG

ATCTATGCATCCTATGCTCTGGCATTTTGGTATGGGACCTCGTTGGTCATATCACGGGAA

TATTCTATTGGACAAGTGCTCACTGTCTTCTTTTCTGTATTAATTGGAGCTTTTAGTATT

GGACAGGCATCTCCGAATATTGAAGCATTTGCAAATGCAAGAGGAGCAGCTTATGAAGTC

TTCAAGATAATTGATAATGAGCCACTCATTGACAGCTTTTCAACAACTGGCCACAAACCT

GAGAATATTAAGGGAAATTTGGAATTTACAAATATTCACTTCAGTTACCCCTCCCGCAAA

GAAGTCGAGATCTTGAAGGGCCTCAACCTGAAGGTGCAGAGCGGGCAGACTGTGGCCCTG

GTTGGGAACAGCGGCTGTGGGAAGAGTACCACGGTGCAGCTGTTGCAGAGGCTCTACGAC

CCCACCGAGGGCACGGTCACTATTGATGGCCAGGACATCAGGACCATAAACGTGAGGTAT

CTGCGGGAGATTATCGGTGTGGTGAGTCAGGAACCTGTGCTGTTTGCCACCACAATAGCT

GAAAACATTCGCTATGGTCGAGAGAATGTCACCATGGAAGAGATTGAGAAAGCTGTCAAG

GAAGCTAACGCCTATGACTTCATCATGAAGCTGCCTCATAAATTCGACACCCTGGTTGGA

GAGAGAGGGGCACAGCTGAGTGGTGGGCAGAAGCAGAGAATCGCCATAGCGCGGGCCCTG

GTTCGCAACCCCAAGATCCTGCTGCTGGACGAGGCCACGTCGGCCCTGGACACGGAGAGT

GAGGCGGTGGTGCAGGTGGCCCTGGACAAGGCCCGAGAAGGCCGGACCACCATTGTGATA

GCTCATCGCCTGTCCACAGTTCGCAACGCCGATGTCATTGCTGGGTTTGAGGATGGAGTC

ATTGTGGAGAGAGGAAATCATGAGGAACTCATGAAAGAGAAAGGCATTTACTACAAACTT

GTCATGATGCAGACAAGAGGAAATGAAATTGAAGTAGAAAATGAAATTTTGGAATCCAAA

AATGAAGTTGATGGGTTGGGATCTTTAAAAGATTCAAGATCCAGTCTAAGAAGAAGATCA

ACTCGCAGCAGTATGCGTGGATCACAGGTCCAAGACAGAAGGCTTAATGCAACAGATGAG

CTGGTTGAAAATGTACCTCCAGTTTCCTTTTGGAGGATTCTAAAGCTGAATTTAACAGAA

TGGCCTTATTTTGTGGTGGGCGTGATTTGTGCCATTATAAACGGAGGCCTGCAACCAGCA

TTTGCAGTGATATTTTCAAGGATTATAGGGATTTTTGCAAGACCCGATGATGTTGAAACA

AAACGTCAGAACAGTCATCTGTTTTCACTGTTGTTTCTAATCCTTGGAATTGTCTCTTTT

GTTACCTTTTTCCTTCAGGGCTTCACATTTGGCAAAGCTGGAGAGATTCTCACCAAGCGG

CTACGATACCTGGTTTTCAGGTCCATGCTGAGACAGGATGTGAGCTGGTTTGATGACCCT

AAAAACACCACAGGAGCACTGACTACCAGGCTTGCTAATGATGCTGCTCAAGTAAAAGGG

GCTATCGGTGCCAGGCTTGCTGTGATTACCCAAAATGTAGCAAATCTTGGGACAGGAATT

ATTATTTCCTTCATCTATGGCTGGCAGTTGACACTTTTACTCTTGGCAATTGTTCCCATC

ATTGCCGTAGCAGGAGTCGTTGAGATGAAAATGCTGTCTGGTAGTGCAATCAAAGATAAG

AAAGAGCTGGAAGGTTCTGGGAAGATCGCTACCGAAGCAATAGAAAACTTCCGAACCGTC

GTGTCTCTGACTCGAGAGGAGAAGTTTGAACACATGTATGGGCAGAGCTTGCGGGTGCCA

TACAGAAACTCTTTACGGAAAGCACACATCTTTGGAATCACCTTTGCCTTCACCCAGGCC

ATGATGTATTTTTCTTATGCTGCTTGTTTCCGGTTTGGTGCCTTCTTGGTGGCACGTAAT

CACATGGAGTTTCAAGATGTTCTGTTGGTATTTTCAGCCATCGTCTTTGGTGCCATGGCA

GTCGGGCAGGTCAGTTCATTTGCCCCTGACTATGCCAAAGCCAAAGTGTCAGCCTCCCAC

ATCATCATGATCATGGAAAAGGTCCCTACCATTGACAGCTACAGCACAGAAGGCCTGAAG

CCGGATATGCTGGAAGGAAATGTGACCTTCAGCAATGTTGTGTTCAACTATCCGACCCGA

CCAGACATCCCAGTGCTTCAGGGGCTGAACCTGCAGGTGAAGAAGGGCCAGACGCTGGCC

TTGGTGGGCAGCAGTGGCTGTGGGAAGAGCACAACAGTCCAGCTTCTGGAGCGGTTCTAC

AACCCCATTTCAGGGACAGTGTTTGTGGATGGCAAAGAAATACAGCAACTGAATGTGCAG

TGGCTGCGAGCACAGCTGGGCATCGTGTCCCAGGAGCCCATCCTGTTTGACTGCAGCATC

GGGGAGAACATCGCCTATGGAGACAACAGCCGGACCGTGTCACAGGAGGAGATCGAGCAG

GCAGCCAGGGAGGCCAACATCCACCAGTTCATCGAGTCGCTGCCTAACAAATATGACACC

AGAGTGGGAGACAAAGGAACCCAGCTCTCTGGAGGCCAGAAACAGCGCATTGCCATTGCT

CGTGCCCTCATCAGGCAGCCTCGGATTTTGCTTTTGGATGAAGCAACATCAGCTCTGGAT

ACCGAAAGTGAAAAGGTTGTCCAGGAAGCCCTTGACAAAGCCAGAGAGGGCCGAACCTGC

ATCGTGATTGCTCATCGCCTCTCCACCATCCAGAATGCAGACCTCATCGTGGTGATTCAG

AACGGCAAGGTCCAGGAGCATGGCACACACCAGCAGCTGATCGCCCAGAAAGGCATCTAT

TTCTCCATGGTCAGGGTCCAGGCTGGAGCAAGGCGC**TAG**TGAAGCCTGCCTATATGGGAT

CGTAAATATTAAATAGTTGTGTTTAAATATAATATTTGATCAGATCAAAAAACAAGAACT

TACTGGATTGGGTAGTTACATATTTAACATTTCCTGCCATATTTAAAGATAATTTCAAGT

GCAGCATCTTCAGAGACTTTGTAATTAAAGGATCATAAATAGAACCATTATCAAATGGAG

AGTGGATGGTAGTTTTAATTGTATTACAGAATTCATAAACGATTTGATGTAATGTGTAAT

TTATGTTTGTAATATGAACTATAACTGACTATTGGTAAAAAGATTACATGAATAACAAAA

TGTACTGTTATGTTTGCCAAAAGTGCCTACAATAAAACTACACTTTCATATGCTTGGGGC

CATATTTTGTAAAATGCATATTTTCTAAAATATATGTGTTTTTGCAATTTGAATAGTATA

ATCTTGGACCTAAACTAGACATGTCCAGAGATCTTTACCAAGACATATTAGATTCAAACT

GTCACAAGTTCAAAACAGAGAATTCTAAAAACCACAAGAGAAAACAAAACACCAAGGTGC

ATTCAGAGTTATGCCCATTCAACTAATAGAATGATTGTGCCAAGACTTACAGAGAAAGGA

ATGGGATGATACATGCAAGGACTCAGTGTAACAACCAACCAAGAATACTATAGCCAATCA

AACTGTTCTTCATGATCGAGCAAATAATAAAGACCTTCTGAGACAAACTGAGACTGAGAT

AATTTATCACCATCAGATGAGCCATACGAAATATGCTTAAGAATAAGGTCCATCATTAAA

ACACATGAATGTCTAAAACTCACATTAATAATTAATCATGTTTGACGACGAGGGAGTTGA

CAAACACCCTGAAAATTAGTAAAGCGCATTTACAACCGTGTCTGCGAGAATGTTTCAGGA

GACACCTGAGCCAGGACCCTGACAGGAGACTTTCCTTAATGTGGACAGCATCATCCACTG

GGAGAAGGAGGATGCCTGCTAGCACAAGCCCTCAGACTCTCTCGTCCTTTCCGCTTCCTA

ACTGCGAAGAAGTGAATATTGCTTCTGCCATGCATTGCCATCATATGGTTCTGGTGCACT

ACCTGTTGGTTTTGGATTGCAACCAAGCCAAGGTGGACTTTTCCTCTTTTAAGAAAGAGG

AACTTTTCCTCTTTTCATCAAGTAATTTGTCAGTTTTGGAAAGTTGACACACATAACCCA

TTACAAGAGTAAATAGTCACAAATTTTAAAGAAACATTACAAGTAACCACCAACCAATAA

AGATAACAGAAGAAGAAAAGGTATAAAGGATCTCTGGGGCATAAGAAAATAAATGATAGA

AGTAATTACCTACCAATAATATCCATGAATAATATCCATGAATGGACTAATTCTCCCAAT

TAAAAGATACAAACTGACTGCAGGGGGAGGCGGGGAAACCAACAATATATTACCTGTAAT

GAAACTTGCTTCACTGGAAGAGACTGAAAGTGAAAGGATTCTGTGCAAATGGAAACCCAT

AACAAGCAGCAGTATAGCTCTACTTTTCAGACAAAATAGATTTAAAAGTTAAATAGAGAC

AAAGGTCATTATATAATGGTCAAAGTACTAATAAGATATGCTGATTGAAAATGGACATGA

ACTTAATGTTGGAATAATTAGTTTTATTACATCTAAAGGAAAATATAGTATATAGCTTGT

GTCTAGATGTTCTTCCAAAGCCTTGTGCGGTCATCACATGGTCACAGGTGGAACTTTTTC

AGAAGTGGCTGGATTTAGGGTATGTGGTGCTGGGACTAAAGGTGTCTGATTACTAAATTA

GTCAACTGATTAATTTGGCACAGTTCTGGGAGGTGCCCCACCCTAAATGTGAGTGGATAC

AATTTAAAGGAACAGAAAGATCATTTGTTGCTTGCTGCTTGGTGAGGGACTTCTGATTGC

CCTAATTTGGCACAGAGTGTTTTTCCTCTACAATGCCCCTCTGCCATGCTGCTCTGGCTT

GGAGCCAGCCAACTATGAACTGAAACCTCTACAAACTATGAGCAAAATAAACTTTTCCTC

CTTTAACTTTATATGTCAGCTATTTTGTTTCAGCAATGAGAAAAGTAACCAAGACAAGGC

TCCAAAGCAGTAACAGTGGAGGATTTCAATGACCACACTTTCATCAATGGATAAATCATT

CAGGCAATCAGCAGGTGCATTATAGTTAAATTGTACTATAGTCTGCTTGTACCTAAAGGA

CTTGTACAGAACATTTCATCCATGAACTAAAGAACGCACATTCTTCTCATCAACATGTGG

AACATTCCATAAACTATATTTTGGGTTGCAGAATTAATCTTAATAAATTAAAAAATTGAA

AACTTGTAACTTATTTGACCACCAAGGAATAAAACTAGAACTCAACAACTAAAGAAACTT

CAGCAATCAAACAAGTATATGGAGACCAAGCAACATATTTTTAAACAAAAAATAGATCAA

GGAAATAAGAAAGGAAATGTAAAAATTTCTCAAAAAATAAAAATGGAAATACAGCATACC

AAAAATTATGGGACACAGCAAAAGCAGTGCCAAAAGTNNGTCNTATNNGTGTATCATCAA

ATTATTCGTGCATGGATGATGAAGATGATCCATTATGGTAAAACATGCTTTTATAACAGA

AGAGTGGTGTGGGTGTATCATGCTGATGATTGATTTTTATGTTGGAC

> Isoform 2 CDS

ATGGATCACGAAGGGGACTTCGGTGGAAAAGCAGGGGACTATAAACTGGGGAAGAAAAGT

AAAAAGGAGAAGAAGGAAAAGAAACCAACTGTTAGTACATTTGCCATGTTTCGCTATTCA

AATTGGCTGGACAGGTTGTACATGGTGCTGGGGACTCTGGCTGCTATCATCCATGGAGCT

GCGCTCCCTCTCTTGATGCTGGTGTTTGGAGACATGACAGACACCTTTGTAAATGGAAGT

GGCACAAACTCATCAAATGCATCAAGTACATTAGATAAAAGTGAAGTCAGTGGACCAGAC

AATCTGGAGGAAAAAATGACCACGTATGCCTATTATTATTCTGGAATCGGTGCTGGTGTG

CTTATTGCTGCTTACATTCAGGTTTCATTTTGGTGCCTGGCAGCTGGAAGACAGATACAC

AAAATTAGAACACAGTTTTTCCATGCAATAATGAAACAGGAGATTGGCTGGTTTGATGTG

CATGATGCTGGGGAGCTGAACACCCGCCCCACAGATGATGTCTCCAAAATTAATGAAGGA

ATTGGTGACAAAATTGGATTGTTCTTTCAATCATTGGCAACATTTTTGACTGGTTTCATA

ATAGGATTTACACGTGGTTGGAAGCTCACTCTTGTGATTTTGGCTGTCAGCCCTGTTCTT

GGACTATCAGCTGCAATCTGGGCAAAGATACTGTCTTCATTTACTGATAAAGAGCTATCA

GCATATGCAAAAGCTGGAGCCGTGGCTGAAGAAGCCTTAGCAGCCATTAGAACTGTGATT

GCATTTGGAGGACAAAGCAAAGAACTTGAAAGGTACAATAAAAATTTAGAAGAAGCTAAA

AGAATTGGAATAAAGAAAGCCATCACATCCAATATTTCTATTGGTGCTGCTTTCCTGCTG

ATCTATGCATCCTATGCTCTGGCATTTTGGTATGGGACCTCGTTGGTCATATCACGGGAA

TATTCTATTGGACAAGTGCTCACTGTCTTCTTTTCTGTATTAATTGGAGCTTTTAGTATT

GGACAGGCATCTCCGAATATTGAAGCATTTGCAAATGCAAGAGGAGCAGCTTATGAAGTC

TTCAAGATAATTGATAATGAGCCACTCATTGACAGCTTTTCAACAACTGGCCACAAACCT

GAGAATATTAAGGGAAATTTGGAATTTACAAATATTCACTTCAGTTACCCCTCCCGCAAA

GAAGTCGAGATCTTGAAGGGCCTCAACCTGAAGGTGCAGAGCGGGCAGACTGTGGCCCTG

GTTGGGAACAGCGGCTGTGGGAAGAGTACCACGGTGCAGCTGTTGCAGAGGCTCTACGAC

CCCACCGAGGGCACGGTCACTATTGATGGCCAGGACATCAGGACCATAAACGTGAGGTAT

CTGCGGGAGATTATCGGTGTGGTGAGTCAGGAACCTGTGCTGTTTGCCACCACAATAGCT

GAAAACATTCGCTATGGTCGAGAGAATGTCACCATGGAAGAGATTGAGAAAGCTGTCAAG

GAAGCTAACGCCTATGACTTCATCATGAAGCTGCCTCATAAATTCGACACCCTGGTTGGA

GAGAGAGGGGCACAGCTGAGTGGTGGGCAGAAGCAGAGAATCGCCATAGCGCGGGCCCTG

GTTCGCAACCCCAAGATCCTGCTGCTGGACGAGGCCACGTCGGCCCTGGACACGGAGAGT

GAGGCGGTGGTGCAGGTGGCCCTGGACAAGGCCCGAGAAGGCCGGACCACCATTGTGATA

GCTCATCGCCTGTCCACAGTTCGCAACGCCGATGTCATTGCTGGGTTTGAGGATGGAGTC

ATTGTGGAGAGAGGAAATCATGAGGAACTCATGAAAGAGAAAGGCATTTACTACAAACTT

GTCATGATGCAGACAAGAGGAAATGAAATTGAAGTAGAAAATGAAATTTTGGAATCCAAA

AATGAAGTTGATGGGTTGGGATCTTTAAAAGATTCAAGATCCAGTCTAAGAAGAAGATCA

ACTCGCAGCAGTATGCGTGGATCACAGGTCCAAGACAGAAGGCTTAATGCAACAGATGAG

CTGGTTGAAAATGTACCTCCAGTTTCCTTTTGGAGGATTCTAAAGCTGAATTTAACAGAA

TGGCCTTATTTTGTGGTGGGCGTGATTTGTGCCATTATAAACGGAGGCCTGCAACCAGCA

TTTGCAGTGATATTTTCAAGGATTATAGGGATTTTTGCAAGACCCGATGATGTTGAAACA

AAACGTCAGAACAGTCATCTGTTTTCACTGTTGTTTCTAATCCTTGGAATTGTCTCTTTT

GTTACCTTTTTCCTTCAGGGCTTCACATTTGGCAAAGCTGGAGAGATTCTCACCAAGCGG

CTACGATACCTGGTTTTCAGGTCCATGCTGAGACAGGATGTGAGCTGGTTTGATGACCCT

AAAAACACCACAGGAGCACTGACTACCAGGCTTGCTAATGATGCTGCTCAAGTAAAAGGG

GCTATCGGTGCCAGGCTTGCTGTGATTACCCAAAATGTAGCAAATCTTGGGACAGGAATT

ATTATTTCCTTCATCTATGGCTGGCAGTTGACACTTTTACTCTTGGCAATTGTTCCCATC

ATTGCCGTAGCAGGAGTCGTTGAGATGAAAATGCTGTCTGGTAGTGCAATCAAAGATAAG

AAAGAGCTGGAAGGTTCTGGGAAGATCGCTACCGAAGCAATAGAAAACTTCCGAACCGTC

GTGTCTCTGACTCGAGAGGAGAAGTTTGAACACATGTATGGGCAGAGCTTGCGGGTGCCA

TACAGAAACTCTTTACGGAAAGCACACATCTTTGGAATCACCTTTGCCTTCACCCAGGCC

ATGATGTATTTTTCTTATGCTGCTTGTTTCCGGTTTGGTGCCTTCTTGGTGGCACGTAAT

CACATGGAGTTTCAAGATGTTCTGTTGGTATTTTCAGCCATCGTCTTTGGTGCCATGGCA

GTCGGGCAGGTCAGTTCATTTGCCCCTGACTATGCCAAAGCCAAAGTGTCAGCCTCCCAC

ATCATCATGATCATGGAAAAGGTCCCTACCATTGACAGCTACAGCACAGAAGGCCTGAAG

CCGGATATGCTGGAAGGAAATGTGACCTTCAGCAATGTTGTGTTCAACTATCCGACCCGA

CCAGACATCCCAGTGCTTCAGGGGCTGAACCTGCAGGTGAAGAAGGGCCAGACGCTGGCC

TTGGTGGGCAGCAGTGGCTGTGGGAAGAGCACAACAGTCCAGCTTCTGGAGCGGTTCTAC

AACCCCATTTCAGGGACAGTGTTTGTGGATGGCAAAGAAATACAGCAACTGAATGTGCAG

TGGCTGCGAGCACAGCTGGGCATCGTGTCCCAGGAGCCCATCCTGTTTGACTGCAGCATC

GGGGAGAACATCGCCTATGGAGACAACAGCCGGACCGTGTCACAGGAGGAGATCGAGCAG

GCAGCCAGGGAGGCCAACATCCACCAGTTCATCGAGTCGCTGCCTAACAAATATGACACC

AGAGTGGGAGACAAAGGAACCCAGCTCTCTGGAGGCCAGAAACAGCGCATTGCCATTGCT

CGTGCCCTCATCAGGCAGCCTCGGATTTTGCTTTTGGATGAAGCAACATCAGCTCTGGAT

ACCGAAAGTGAAAAGGTTGTCCAGGAAGCCCTTGACAAAGCCAGAGAGGGCCGAACCTGC

ATCGTGATTGCTCATCGCCTCTCCACCATCCAGAATGCAGACCTCATCGTGGTGATTCAG

AACGGCAAGGTCCAGGAGCATGGCACACACCAGCAGCTGATCGCCCAGAAAGGCATCTAT

TTCTCCATGGTCAGGGTCCAGGCTAGAGCAAAGACCTAG

>Isoform 3 CDS

ATGGATCACGAAGGGGACTTCGGTGGAAAAGCAGGGGACTATAAACTGGGGAAGAAAAGT

AAAAAGGAGAAGAAGGAAAAGAAACCAACTGTTAGTACATTTGCCATGTTTCGCTATTCA

AATTGGCTGGACAGGTTGTACATGGTGCTGGGGACTCTGGCTGCTATCATCCATGGAGCT

GCGCTCCCTCTCTTGATGCTGGTGTTTGGAGACATGACAGACACCTTTGTAAATGGAAGT

GGCACAAACTCATCAAATGCATCAAGTACATTAGATAAAAGTGAAGTCAGTGGACCAGAC

AATCTGGAGGAAAAAATGACCACGTATGCCTATTATTATTCTGGAATCGGTGCTGGTGTG

CTTATTGCTGCTTACATTCAGGTTTCATTTTGGTGCCTGGCAGCTGGAAGACAGATACAC

AAAATTAGAACACAGTTTTTCCATGCAATAATGAAACAGGAGATTGGCTGGTTTGATGTG

CATGATGCTGGGGAGCTGAACACCCGCCCCACAGATGATGTCTCCAAAATTAATGAAGGA

ATTGGTGACAAAATTGGATTGTTCTTTCAATCATTGGCAACATTTTTGACTGGTTTCATA

ATAGGATTTACACGTGGTTGGAAGCTCACTCTTGTGATTTTGGCTGTCAGCCCTGTTCTT

GGACTATCAGCTGCAATCTGGGCAAAGATACTGTCTTCATTTACTGATAAAGAGCTATCA

GCATATGCAAAAGCTGGAGCCGTGGCTGAAGAAGCCTTAGCAGCCATTAGAACTGTGATT

GCATTTGGAGGACAAAGCAAAGAACTTGAAAGGTACAATAAAAATTTAGAAGAAGCTAAA

AGAATTGGAATAAAGAAAGCCATCACATCCAATATTTCTATTGGTGCTGCTTTCCTGCTG

ATCTATGCATCCTATGCTCTGGCATTTTGGTATGGGACCTCGTTGGTCATATCACGGGAA

TATTCTATTGGACAAGTGCTCACTGTCTTCTTTTCTGTATTAATTGGAGCTTTTAGTATT

GGACAGGCATCTCCGAATATTGAAGCATTTGCAAATGCAAGAGGAGCAGCTTATGAAGTC

TTCAAGATAATTGATAATGAGCCACTCATTGACAGCTTTTCAACAACTGGCCACAAACCT

GAGAATATTAAGGGAAATTTGGAATTTACAAATATTCACTTCAGTTACCCCTCCCGCAAA

GAAGTCGAGATCTTGAAGGGCCTCAACCTGAAGGTGCAGAGCGGGCAGACTGTGGCCCTG

GTTGGGAACAGCGGCTGTGGGAAGAGTACCACGGTGCAGCTGTTGCAGAGGCTCTACGAC

CCCACCGAGGGCACGGTCACTATTGATGGCCAGGACATCAGGACCATAAACGTGAGGTAT

CTGCGGGAGATTATCGGTGTGGTGAGTCAGGAACCTGTGCTGTTTGCCACCACAATAGCT

GAAAACATTCGCTATGGTCGAGAGAATGTCACCATGGAAGAGATTGAGAAAGCTGTCAAG

GAAGCTAACGCCTATGACTTCATCATGAAGCTGCCTCATAAATTCGACACCCTGGTTGGA

GAGAGAGGGGCACAGCTGAGTGGTGGGCAGAAGCAGAGAATCGCCATAGCGCGGGCCCTG

GTTCGCAACCCCAAGATCCTGCTGCTGGACGAGGCCACGTCGGCCCTGGACACGGAGAGT

GAGGCGGTGGTGCAGGTGGCCCTGGACAAGGCCCGAGAAGGCCGGACCACCATTGTGATA

GCTCATCGCCTGTCCACAGTTCGCAACGCCGATGTCATTGCTGGGTTTGAGGATGGAGTC

ATTGTGGAGAGAGGAAATCATGAGGAACTCATGAAAGAGAAAGGCATTTACTACAAACTT

GTCATGATGCAGACAAGAGGAAATGAAATTGAAGTAGAAAATGAAATTTTGGAATCCAAA

AATGAAGTTGATGGGTTGGGATCTTTAAAAGATTCAAGATCCAGTCTAAGAAGAAGATCA

ACTCGCAGCAGTATGCGTGGATCACAGGTCCAAGACAGAAGGCTTAATGCAACAGATGAG

CTGGTTGAAAATGTACCTCCAGTTTCCTTTTGGAGGATTCTAAAGCTGAATTTAACAGAA

TGGCCTTATTTTGTGGTGGGCGTGATTTGTGCCATTATAAACGGAGGCCTGCAACCAGCA

TTTGCAGTGATATTTTCAAGGATTATAGGGATTTTTGCAAGACCCGATGATGTTGAAACA

AAACGTCAGAACAGTCATCTGTTTTCACTGTTGTTTCTAATCCTTGGAATTGTCTCTTTT

GTTACCTTTTTCCTTCAGGGCTTCACATTTGGCAAAGCTGGAGAGATTCTCACCAAGCGG

CTACGATACCTGGTTTTCAGGTCCATGCTGAGACAGGATGTGAGCTGGTTTGATGACCCT

AAAAACACCACAGGAGCACTGACTACCAGGCTTGCTAATGATGCTGCTCAAGTAAAAGGG

GCTATCGGTGCCAGGCTTGCTGTGATTACCCAAAATGTAGCAAATCTTGGGACAGGAATT

ATTATTTCCTTCATCTATGGCTGGCAGTTGACACTTTTACTCTTGGCAATTGTTCCCATC

ATTGCCGTAGCAGGAGTCGTTGAGATGAAAATGCTGTCTGGTAGTGCAATCAAAGATAAG

AAAGAGCTGGAAGGTTCTGGGAAGATCGCTACCGAAGCAATAGAAAACTTCCGAACCGTC

GTGTCTCTGACTCGAGAGGAGAAGTTTGAACACATGTATGGGCAGAGCTTGCGGGTGCCA

TACAGAAACTCTTTACGGAAAGCACACATCTTTGGAATCACCTTTGCCTTCACCCAGGCC

ATGATGTATTTTTCTTATGCTGCTTGTTTCCGGTTTGGTGCCTTCTTGGTGGCACGTAAT

CACATGGAGTTTCAAGATGTTCTGTTGGTATTTTCAGCCATCGTCTTTGGTGCCATGGCA

GTCGGGCAGGTCAGTTCATTTGCCCCTGACTATGCCAAAGCCAAAGTGTCAGCCTCCCAC

ATCATCATGATCATGGAAAAGGTCCCTACCATTGACAGCTACAGCACAGAAGGCCTGAAG

CCGGATATGCTGGAAGGAAATGTGACCTTCAGCAATGTTGTGTTCAACTATCCGACCCGA

CCAGACATCCCAGTGCTTCAGGGGCTGAACCTGCAGGTGAAGAAGGGCCAGACGCTGGCC

TTGGTGGGCAGCAGTGGCTGTGGGAAGAGCACAACAGTCCAGCTTCTGGAGCGGTTCTAC

AACCCCATTTCAGGGACAGTGTTTGTGGATGGCAAAGAAATACAGCAACTGAATGTGCAG

TGGCTGCGAGCACAGCTGGGCATCGTGTCCCAGGAGCCCATCCTGTTTGACTGCAGCATC

GGGGAGAACATCGCCTATGGAGACAACAGCCGGACCGTGTCACAGGAGGAGATCGAGCAG

GCAGCCAGGGAGGCCAACATCCACCAGTTCATCGAGTCGCTGCCTAACAAATATGACACC

AGAGTGGGAGACAAAGGAACCCAGCTCTCTGGAGGCCAGAAACAGCGCATTGCCATTGCT

CGTGCCCTCATCAGGCAGCCTCGGATTTTGCTTTTGGATGAAGCAACATCAGCTCTGGAT

ACCGAAAGTGAAAAGGTTGTCCAGGAAGCCCTTGACAAAGCCAGAGAGGGCCGAACCTGC

ATCGTGATTGCTCATCGCCTCTCCACCATCCAGAATGCAGACCTCATCGTGGTGATTCAG

AACGGCAAGGTCCAGGAGCATGGCACACACCAGCAGCTGATCGCCCAGAAAGGCATCTAT

TTCTCCATGGTCAGGGTCCAGGCTGGAGCAAGGCGCTAGTGAAGCCTGCCTATATGGGAT

CGTAAATATTAAATAGTTGTGTTTAAATATAATATTTGATCAGATCAAAAAACAAGAACT

TACTGGATTGGGTAGTTACATATTTAACATTTCCTGCCATATTTAAAGATAATTTCAAGT

GCAGCATCTTCAGAGACTTTGTAATTAAAGGATCATAAATAGAACCATTATCAAATGGAG

AGTGGATGGTAGTTTTAATTGTATTACAGAATTCATAAACGATTTGATGTAATGTGTAAT

TTATGTTTGTAATATGAACTATAACTGACTATTGGTAAAAAGATTACATGAATAACAAAA

TGTACTGTTATGTTTGCCAAAAGTGCCT

**Table S4.** Guinea pig Abcb1 protein sequences.

>Isoforms 1 and 3

MDHEGDFGGKAGDYKLGKKSKKEKKEKKPTVSTFAMFRYSNWLDRLYMVLGTLAAIIHGA

ALPLLMLVFGDMTDTFVNGSGTNSSNASSTLDKSEVSGPDNLEEKMTTYAYYYSGIGAGV

LIAAYIQVSFWCLAAGRQIHKIRTQFFHAIMKQEIGWFDVHDAGELNTRPTDDVSKINEG

IGDKIGLFFQSLATFLTGFIIGFTRGWKLTLVILAVSPVLGLSAAIWAKILSSFTDKELS

AYAKAGAVAEEALAAIRTVIAFGGQSKELERYNKNLEEAKRIGIKKAITSNISIGAAFLL

IYASYALAFWYGTSLVISREYSIGQVLTVFFSVLIGAFSIGQASPNIEAFANARGAAYEV

FKIIDNEPLIDSFSTTGHKPENIKGNLEFTNIHFSYPSRKEVEILKGLNLKVQSGQTVAL

VGNSGCGKSTTVQLLQRLYDPTEGTVTIDGQDIRTINVRYLREIIGVVSQEPVLFATTIA

ENIRYGRENVTMEEIEKAVKEANAYDFIMKLPHKFDTLVGERGAQLSGGQKQRIAIARAL

VRNPKILLLDEATSALDTESEAVVQVALDKAREGRTTIVIAHRLSTVRNADVIAGFEDGV

IVERGNHEELMKEKGIYYKLVMMQTRGNEIEVENEILESKNEVDGLGSLKDSRSSLRRRS

TRSSMRGSQVQDRRLNATDELVENVPPVSFWRILKLNLTEWPYFVVGVICAIINGGLQPA

FAVIFSRIIGIFARPDDVETKRQNSHLFSLLFLILGIVSFVTFFLQGFTFGKAGEILTKR

LRYLVFRSMLRQDVSWFDDPKNTTGALTTRLANDAAQVKGAIGARLAVITQNVANLGTGI

IISFIYGWQLTLLLLAIVPIIAVAGVVEMKMLSGSAIKDKKELEGSGKIATEAIENFRTV

VSLTREEKFEHMYGQSLRVPYRNSLRKAHIFGITFAFTQAMMYFSYAACFRFGAFLVARN

HMEFQDVLLVFSAIVFGAMAVGQVSSFAPDYAKAKVSASHIIMIMEKVPTIDSYSTEGLK

PDMLEGNVTFSNVVFNYPTRPDIPVLQGLNLQVKKGQTLALVGSSGCGKSTTVQLLERFY

NPISGTVFVDGKEIQQLNVQWLRAQLGIVSQEPILFDCSIGENIAYGDNSRTVSQEEIEQ

AAREANIHQFIESLPNKYDTRVGDKGTQLSGGQKQRIAIARALIRQPRILLLDEATSALD

TESEKVVQEALDKAREGRTCIVIAHRLSTIQNADLIVVIQNGKVQEHGTHQQLIAQKGIY

FSMVRVQAGARR-

>Isoform 2

MDHEGDFGGKAGDYKLGKKSKKEKKEKKPTVSTFAMFRYSNWLDRLYMVLGTLAAIIHGA

ALPLLMLVFGDMTDTFVNGSGTNSSNASSTLDKSEVSGPDNLEEKMTTYAYYYSGIGAGV

LIAAYIQVSFWCLAAGRQIHKIRTQFFHAIMKQEIGWFDVHDAGELNTRPTDDVSKINEG

IGDKIGLFFQSLATFLTGFIIGFTRGWKLTLVILAVSPVLGLSAAIWAKILSSFTDKELS

AYAKAGAVAEEALAAIRTVIAFGGQSKELERYNKNLEEAKRIGIKKAITSNISIGAAFLL

IYASYALAFWYGTSLVISREYSIGQVLTVFFSVLIGAFSIGQASPNIEAFANARGAAYEV

FKIIDNEPLIDSFSTTGHKPENIKGNLEFTNIHFSYPSRKEVEILKGLNLKVQSGQTVAL

VGNSGCGKSTTVQLLQRLYDPTEGTVTIDGQDIRTINVRYLREIIGVVSQEPVLFATTIA

ENIRYGRENVTMEEIEKAVKEANAYDFIMKLPHKFDTLVGERGAQLSGGQKQRIAIARAL

VRNPKILLLDEATSALDTESEAVVQVALDKAREGRTTIVIAHRLSTVRNADVIAGFEDGV

IVERGNHEELMKEKGIYYKLVMMQTRGNEIEVENEILESKNEVDGLGSLKDSRSSLRRRS

TRSSMRGSQVQDRRLNATDELVENVPPVSFWRILKLNLTEWPYFVVGVICAIINGGLQPA

FAVIFSRIIGIFARPDDVETKRQNSHLFSLLFLILGIVSFVTFFLQGFTFGKAGEILTKR

LRYLVFRSMLRQDVSWFDDPKNTTGALTTRLANDAAQVKGAIGARLAVITQNVANLGTGI

IISFIYGWQLTLLLLAIVPIIAVAGVVEMKMLSGSAIKDKKELEGSGKIATEAIENFRTV

VSLTREEKFEHMYGQSLRVPYRNSLRKAHIFGITFAFTQAMMYFSYAACFRFGAFLVARN

HMEFQDVLLVFSAIVFGAMAVGQVSSFAPDYAKAKVSASHIIMIMEKVPTIDSYSTEGLK

PDMLEGNVTFSNVVFNYPTRPDIPVLQGLNLQVKKGQTLALVGSSGCGKSTTVQLLERFY

NPISGTVFVDGKEIQQLNVQWLRAQLGIVSQEPILFDCSIGENIAYGDNSRTVSQEEIEQ

AAREANIHQFIESLPNKYDTRVGDKGTQLSGGQKQRIAIARALIRQPRILLLDEATSALD

TESEKVVQEALDKAREGRTCIVIAHRLSTIQNADLIVVIQNGKVQEHGTHQQLIAQKGIY

FSMVRVQARAKT-

Table S5. RT-PCR primer pairs used for RT-PCR on hypothetical human *ABCB1* exons 30-32. *Used for positive controls.

| **Set** | **Primer Name** | **Primer sequence (5'-3')** | **Product MW (bp)** |
| --- | --- | --- | --- |
| 1 | Human29F1 | TGCATTGTGATTGCTCACCGCC | 389 |
|  | Human30R1 | GAAAATGTTTCTTAAACATAAGAACTT |  |
| 2 | Human29F1 | TGCATTGTGATTGCTCACCGCC | 1,106 or 386 |
|  | Human31R1 | GATTTAGGGCCCACCAGAATAATC |  |
| 3 | Human29F1 | TGCATTGTGATTGCTCACCGCC | 1,135 or 1,103 or 615 |
|  | Human32R1 | GCAAGTGTTAAGTGGCCTCGAG |  |
| 4 | Human28F1 | CAACGCATTGCCATAGCTCGTG | 689 |
|  | Human30R1 | GAAAATGTTTCTTAAACATAAGAACTT |  |
| 5 | Human28F1 | CAACGCATTGCCATAGCTCGTG | 1,406 or 686 |
|  | Human31R1 | GATTTAGGGCCCACCAGAATAATC |  |
| 6 | Human28F1 | CAACGCATTGCCATAGCTCGTG | 1,635 or 1,403 or 915 |
|  | Human32R1 | GCAAGTGTTAAGTGGCCTCGAG |  |
| 7* | Human8/9F | GCTGTCTGGGCAAAGATACTATCTTCATTT | 155 |
|  | Human9/10R | AAATTTTTGTTGTACCTTTCAAGTTCTTTC |  |
